# Quantitative pathology and *APOE* genotype reveal dementia risk and progression in Lewy body disease

**DOI:** 10.1093/brain/awag114

**Published:** 2026-03-27

**Authors:** Hemanth R Nelvagal, Nancy Chiraki, Toby Curless, Patrick W Cullinane, Alice Rockliffe, Samarth Pimparkar, Haruka Kawamura, Sophie Ollerenshaw, Isha Elahi, Sebastian Brandner, Lesley Wu, Raquel Real, Mina Ryten, John Hardy, Eduardo De Pablo Fernandez, Thomas T Warner, Huw R Morris, Yau Mun Lim, Zane Jaunmuktane

**Affiliations:** Department of Clinical and Movement Neurosciences, UCL Queen Square Institute of Neurology, University College London, London WC1N 3BG, UK; Queen Square Brain Bank for Neurological Disorders, UCL Queen Square Institute of Neurology, London WC1N 1PJ, UK; Aligning Science Across Parkinson’s (ASAP) Collaborative Research Network, Chevy Chase, MD 20815, USA; Department of Clinical and Movement Neurosciences, UCL Queen Square Institute of Neurology, University College London, London WC1N 3BG, UK; Queen Square Brain Bank for Neurological Disorders, UCL Queen Square Institute of Neurology, London WC1N 1PJ, UK; Aligning Science Across Parkinson’s (ASAP) Collaborative Research Network, Chevy Chase, MD 20815, USA; Aligning Science Across Parkinson’s (ASAP) Collaborative Research Network, Chevy Chase, MD 20815, USA; Department of Neuromuscular Diseases, UCL Queen Square Institute of Neurology, University College London, London WC1N 3BG, UK; Department of Clinical and Movement Neurosciences, UCL Queen Square Institute of Neurology, University College London, London WC1N 3BG, UK; Queen Square Brain Bank for Neurological Disorders, UCL Queen Square Institute of Neurology, London WC1N 1PJ, UK; Reta Lila Weston Institute of Neurological Studies, UCL Queen Square Institute of Neurology, London WC1N 3BG, UK; Queen Square Movement Disorders Centre, UCL Queen Square Institute of Neurology, London WC1N 3BG, UK; Department of Clinical and Movement Neurosciences, UCL Queen Square Institute of Neurology, University College London, London WC1N 3BG, UK; Queen Square Brain Bank for Neurological Disorders, UCL Queen Square Institute of Neurology, London WC1N 1PJ, UK; Department of Clinical and Movement Neurosciences, UCL Queen Square Institute of Neurology, University College London, London WC1N 3BG, UK; Queen Square Brain Bank for Neurological Disorders, UCL Queen Square Institute of Neurology, London WC1N 1PJ, UK; Queen Square Brain Bank for Neurological Disorders, UCL Queen Square Institute of Neurology, London WC1N 1PJ, UK; Queen Square Brain Bank for Neurological Disorders, UCL Queen Square Institute of Neurology, London WC1N 1PJ, UK; Queen Square Brain Bank for Neurological Disorders, UCL Queen Square Institute of Neurology, London WC1N 1PJ, UK; Department of Neurodegenerative Disease, UCL Queen Square Institute of Neurology, London WC1N 3BG, UK; Division of Neuropathology, National Hospital for Neurology and Neurosurgery, University College London NHS Foundation Trust, London NW1 2PB, UK; Department of Clinical and Movement Neurosciences, UCL Queen Square Institute of Neurology, University College London, London WC1N 3BG, UK; Department of Clinical and Movement Neurosciences, UCL Queen Square Institute of Neurology, University College London, London WC1N 3BG, UK; Aligning Science Across Parkinson’s (ASAP) Collaborative Research Network, Chevy Chase, MD 20815, USA; Aligning Science Across Parkinson’s (ASAP) Collaborative Research Network, Chevy Chase, MD 20815, USA; UK Dementia Research Institute at The University of Cambridge, Cambridge CB2 0AH, UK; Aligning Science Across Parkinson’s (ASAP) Collaborative Research Network, Chevy Chase, MD 20815, USA; Department of Neurodegenerative Disease, UCL Queen Square Institute of Neurology, London WC1N 3BG, UK; Department of Clinical and Movement Neurosciences, UCL Queen Square Institute of Neurology, University College London, London WC1N 3BG, UK; Queen Square Brain Bank for Neurological Disorders, UCL Queen Square Institute of Neurology, London WC1N 1PJ, UK; Reta Lila Weston Institute of Neurological Studies, UCL Queen Square Institute of Neurology, London WC1N 3BG, UK; Centre for Preventive Neurology, Wolfson Institute of Population Health, Queen Mary University London, London E1 4NS, UK; Department of Clinical and Movement Neurosciences, UCL Queen Square Institute of Neurology, University College London, London WC1N 3BG, UK; Queen Square Brain Bank for Neurological Disorders, UCL Queen Square Institute of Neurology, London WC1N 1PJ, UK; Reta Lila Weston Institute of Neurological Studies, UCL Queen Square Institute of Neurology, London WC1N 3BG, UK; Queen Square Movement Disorders Centre, UCL Queen Square Institute of Neurology, London WC1N 3BG, UK; Department of Clinical and Movement Neurosciences, UCL Queen Square Institute of Neurology, University College London, London WC1N 3BG, UK; Aligning Science Across Parkinson’s (ASAP) Collaborative Research Network, Chevy Chase, MD 20815, USA; Queen Square Movement Disorders Centre, UCL Queen Square Institute of Neurology, London WC1N 3BG, UK; Aligning Science Across Parkinson’s (ASAP) Collaborative Research Network, Chevy Chase, MD 20815, USA; Department of Neurodegenerative Disease, UCL Queen Square Institute of Neurology, London WC1N 3BG, UK; Department of Clinical and Movement Neurosciences, UCL Queen Square Institute of Neurology, University College London, London WC1N 3BG, UK; Queen Square Brain Bank for Neurological Disorders, UCL Queen Square Institute of Neurology, London WC1N 1PJ, UK; Aligning Science Across Parkinson’s (ASAP) Collaborative Research Network, Chevy Chase, MD 20815, USA

**Keywords:** Lewy body diseases, Parkinson’s disease, *APOE* genotype, orthostatic hypotension with ischaemic pathology, Alzheimer’s co-pathology, SuStaIn

## Abstract

Dementia in Lewy body diseases (LBD) is common and arises through heterogeneous and incompletely understood pathways. Evidence suggests contributions from genetic factors, including *APOE* ɛ4 genotype, and co-pathology, including concomitant Alzheimer’s disease pathology and hypoperfusion related to orthostatic hypotension. However, the relative impact of these factors remains unclear.

To address this, we analysed 399 post-mortem brains from LBD cases comprising Parkinson’s disease, Parkinson’s disease dementia and dementia with Lewy bodies, and controls, integrating *APOE* genotype, clinical data and assessment of ischaemic pathology alongside large-scale digital pathology quantification. We established an image analysis pipeline using machine learning to enable automated, standardized measurement of α-synuclein, amyloid-β and phosphorylated tau burden across multiple brain regions.

Quantitative pathology was strongly correlated with semi-quantitative ratings and outperformed conventional staging in predicting dementia. Across multiple analytical approaches, *APOE* ɛ3 and ɛ4 carriers showed distinct dementia risk profiles. *APOE* ɛ3 carriers developed dementia at lower quantitative α-synuclein and amyloid-β thresholds than ɛ4 carriers, although overall dementia risk was dominated by ɛ4 genotype, consistent with ɛ4 both promoting greater pathology accumulation and modifying the threshold for dementia onset. Orthostatic hypotension and ischaemic pathology increased dementia risk only in ɛ3 carriers with low Lewy and Alzheimer’s proteinopathy burden, and male sex further modulated dementia risk for this subgroup. The Subtype and Stage Inference (SuStaIn) algorithm identified four trajectories of Lewy pathology progression. Two corresponded to recognized patterns: (i) brainstem first; and (ii) with early amygdala and concomitant brainstem involvement. Two further patterns showed early cortical involvement: (i) with early cingulate cortex involvement together with brainstem pathology; and (ii) starting in the neocortex before limbic and brainstem involvement. Co-pathology progression modelling identified subtypes with early predominance of amyloid-β, phosphorylated tau or α-synuclein and showed that Lewy subtypes follow two propagation trajectories in opposite directions.

Together, these findings demonstrate that integrating quantitative pathology with genotype and clinical data reveals distinct yet overlapping pathways to dementia in LBD, refining disease progression models and providing a basis for genotype- and pathology-informed patient stratification in therapeutic trials.

## Teaser

Nelvagal *et al.* used machine learning and disease modelling to study 399 brains from people with Lewy body diseases and controls. The findings reveal multiple pathways to dementia shaped by pathology burden, APOE genotype, and clinical factors, helping to refine progression models and provide a basis for precision therapies.

## Introduction

Primary Lewy body diseases (LBD) encompass Parkinson’s disease (PD), Parkinson’s disease dementia (PDD) and dementia with Lewy bodies (DLB). Dementia is common in LBD and arises through poorly understood, heterogeneous pathways. LBD represent a significant clinical and societal burden, with no disease-modifying therapies available. In PD, dementia may emerge late in the disease course, or cognitive symptoms may appear early, before or soon after motor features.^1,2^ The 1 year rule classifying patients as having DLB if dementia occurs before or within 1 year of motor symptom onset, or PDD if dementia develops >1 year after motor onset, is clinically useful but remains arbitrary and without a proven biological basis.

Several genetic and pathological factors have been implicated in LBD dementia risk. High Lewy pathology stages, increased cortical Lewy burden and advancing Alzheimer’s disease (AD) co-pathology are associated with higher dementia risk.^3,4^ However, neuropathological examination alone cannot reliably differentiate PD, PDD and DLB at the individual level, and therefore these conditions are classified under the umbrella of LBD.^2,5^

The *APOE* ɛ4 allele, the major genetic risk factor for AD, is also the strongest established genetic risk factor for DLB,^6^ although its effect is greatest in DLB cases with substantial AD co-pathology.^7^  *APOE* ɛ4 is not associated with risk or age of onset of PD, but is linked with higher cortical Lewy pathology^8^ and increased risk of progression to dementia in PD.^9,10^

AD-type amyloid-β (Aβ) and phosphorylated tau (pTau) co-pathology are common in LBD and influence clinical progression,^4^ with experimental evidence suggesting that Aβ pathology can exacerbate α-synuclein aggregation,^11-13^ supporting the view that these pathologies are biologically intertwined,^14^ although the mechanisms for interaction remain unclear.

In addition to misfolded proteinopathies, orthostatic hypotension (OH) is commonly associated with dementia,^15-17^ but neuropathological confirmation of this relationship is lacking.

Although data-driven disease progression modelling with the Subtype and Stage Inference (SuStaIn) algorithm has been used in population cohorts with pathologically confirmed Lewy pathology,^18^ it has not previously been applied to large, post-mortem primary LBD datasets with quantitative Lewy body, Aβ and pTau pathology.

Overall, the extent to which *APOE* genotype, quantitative regional burden of proteinopathies, OH and ischaemic pathology contribute to dementia risk in LBD remains unclear. To address this, we developed and applied a quantitative digital pathology pipeline to a large, neuropathologically confirmed cohort of primary LBD and controls, generating automated quantitative measures of α-synuclein, Aβ and pTau across multiple brain regions. To capture the multidimensional nature of dementia risk in LBD, we applied a complementary set of analytical approaches. Multivariable logistic regression was used to identify independent clinicopathological predictors of dementia. A rule-based decision-tree approach provided interpretable, clinically applicable stratification based on quantitative pathology and ischaemic factors. Finally, SuStaIn modelling was applied to identify heterogeneity in LBD progression trajectories. The overall objective of this work was to define *APOE*-modulated disease progression patterns and clinicopathological pathways to dementia in LBD.

## Materials and methods

### Study design and participants

This retrospective post-mortem study included neuropathologically confirmed LBD patients with clinical parkinsonism and controls without neurological disease. Cases were selected from the UCL Queen Square Brain Bank for Neurological Disorders (QSBB) archive between 1991 and 2022. Consecutive patients were included unless medical records were incomplete or formalin-fixed paraffin-embedded blocks unavailable. QSBB protocols and this study have been approved by the NHS Health Research Authority Ethics Committee London-Central (REC reference 23/LO/0044). Written informed consent for the use of biological materials and clinical data in medical research was obtained for all cases from the donors or their next of kin.

### Clinical assessment

Medical records were systematically reviewed to extract demographic and clinical information, including age at death, disease duration, sex and dementia status. Dementia was defined as the presence of objective cognitive impairment documented by a clinician or neuropsychological test severe enough to affect tasks of daily living significantly, not attributable to motor impairment.^1^ The temporal relationship between motor and cognitive symptom onset was documented and applied to classify cases as PDD or DLB based on whether dementia developed more or less than 1 year after motor symptom onset.^2^ OH and clinical diagnosis of infarct or haemorrhage were also recorded. OH was defined as: (i) documented drop in systolic/diastolic blood pressure ≥20/10 mmHg on standing^19^; or (ii) presence of postural symptoms suggestive of OH according to the impression of the treating clinician, persistent for >6 months after therapeutic measures to address secondary (non-neurogenic) causes of OH had been implemented.^16^

### Genotyping

DNA was extracted from cerebellar or frontal lobe tissue and genotyped using the Illumina NeuroBooster array,^20^ which provides dense coverage of neurodegenerative disease-related variants. Genotype data were filtered using PLINK v.2.^21^ Individuals and single nucleotide polymorphisms with >5% missing data were excluded using the -mind 0.05 and -geno 0.05 filters. Genotype data were imputed against the TOPMed r2 panel^22^ with Eagle v.2.4^23^ phasing on the TOPMed Imputation Server using Minimac4. *APOE* genotypes were inferred from imputed genotypes at the rs7412 and rs429358 single nucleotide polymorphisms, which define the ɛ2, ɛ3 and ɛ4 alleles. *APOE* ɛ2/ɛ3 and ɛ3/ɛ3 genotypes were classified as ɛ3, whereas ɛ2/ɛ4, ɛ3/ɛ4 and ɛ4/ɛ4 genotypes were classified as ɛ4. Cases carrying known pathogenic mutations in *SNCA*, *PRKN* or *PINK1*, identified through directly genotyped or high-quality imputed variants included in the array and annotated as pathogenic or risk-associated alleles,^24^ were excluded from the study. Variants in other PD-associated risk genes, such as *GBA* and *LRRK2*, and genes linked to AD or other neurodegenerative conditions were not assessed.

### Neuropathological assessment

Across the cohort, the majority of immunostained slides were archival. A subset of cases required new staining because of to missing, damaged or inadequate archival slides. Details of restained slides, immunostaining protocols and analysed brain regions are provided in the Supplementary material. For each case, the extent of pathology was assessed according to standard criteria for Lewy (Braak stage),^25^ Aβ (Thal phase)^26^ and pTau pathology (Braak and Braak stage).^27^ Alzheimer’s disease neuropathological change (ADNC) was determined according to National Institute on Aging–Alzheimer’s Association (NIA-AA) guidelines,^28,29^ and TDP-43 pathology was evaluated using limbic-predominant age-related TDP-43 encephalopathy (LATE) staging.^30^ Pathology records were reviewed for haemorrhagic and ischaemic pathology, defined as macroscopic or microscopic infarcts, or acute ischaemic neuronal eosinophilia, in vascular watershed and other regions, recorded as present or absent.

Haematoxylin and eosin and immunohistochemistry-stained whole slides were digitized on a Hamamatsu NanoZoomer S360 (26 mm × 76 mm slides; 40× objective; JPEG compression quality 70; pixel size 0.23 µm per pixel) or Hamamatsu NanoZoomer S60 (double 52 mm × 76 mm slides; 40× objective; JPEG compression quality 65; pixel size 0.22 µm per pixel) slide scanners (Fig. 1).

**Figure 1:**
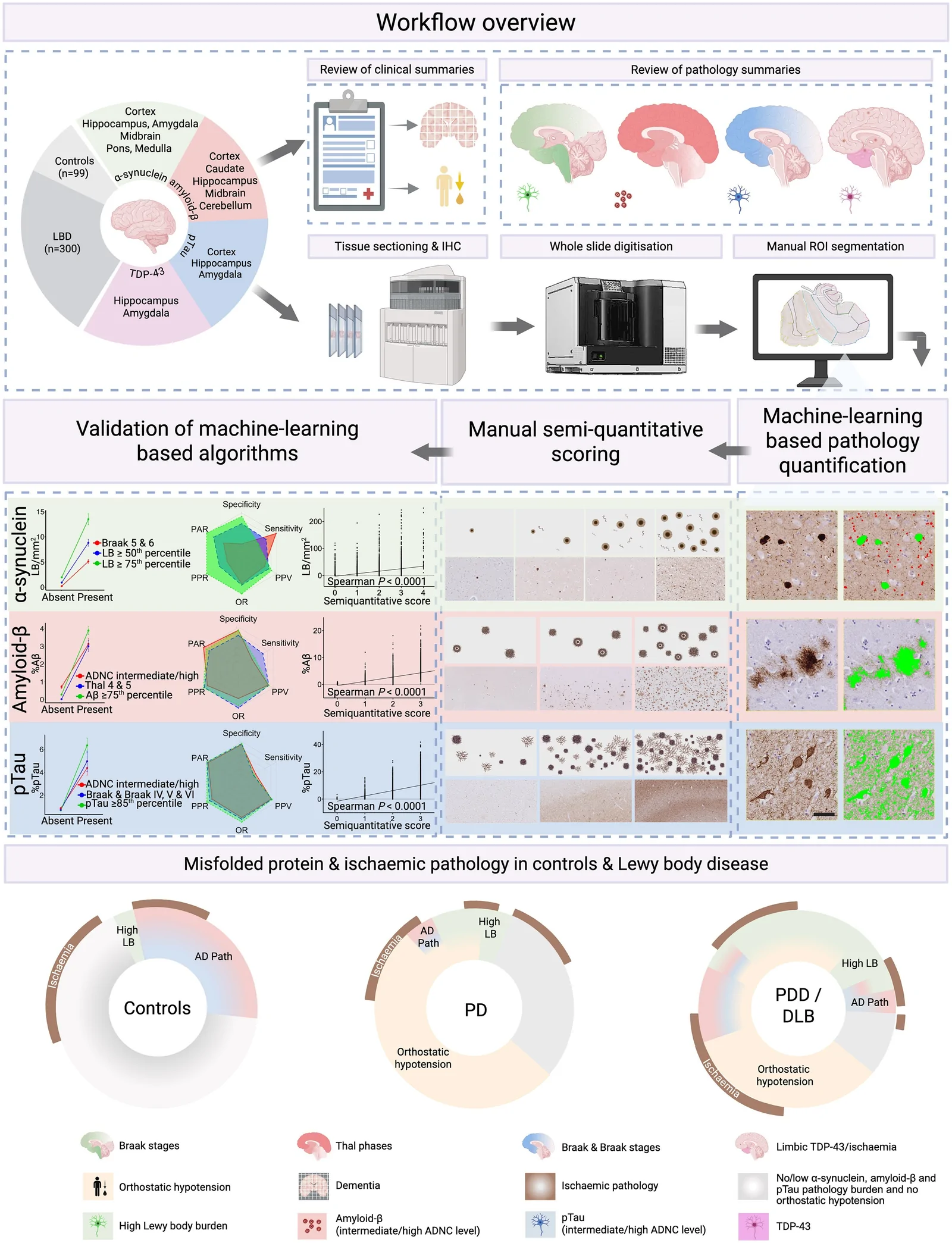
**Cohort design, digital pathology quantification, method validation and clinical correlates.**  *Top*: Overview of the study workflow. The cohort comprised 399 individuals (99 controls; 300 LBD: PD, PDD and DLB). Clinical reports were reviewed to extract demographic variables, dementia status, orthostatic hypotension and vascular pathology, and neuropathology reports were used to derive pathology severity scores and assess the presence of haemorrhagic and ischaemic pathology. Following digitization of immunohistochemically (IHC) stained slides for α-synuclein, amyloid-β (Aβ), phosphorylated tau (pTau) and TDP-43, regions of interest (ROIs) were delineated manually across defined cortical, limbic and brainstem areas. *Middle*, from *right* to *left*: Machine-learning models quantified α-synuclein burden (LB/mm^2^) and Aβ/pTau burden (% area) across each ROI. Representative ×40 images (*left*) and corresponding detected signal (*right*; green = included, red = excluded; scale bar = 100 µm) illustrate the approach. Manual semi-quantitative ratings (α-synuclein, 0–4; Aβ/pTau, 0–3) were assigned in parallel. Quantitative measures showed strong correspondence to semi-quantitative scores across matched ROIs (Spearman’s ρ: α-synuclein = 0.72, Aβ = 0.80, pTau = 0.76; all *P* < 0.0001). Radar plots compare dementia prediction performance for quantitative thresholds (50th, 75th and 85th percentiles) versus traditional staging criteria [Braak Lewy stages, Thal phases, Braak and Braak pTau stages and Alzheimer’s disease neuropathological change (ADNC)]. Metrics include sensitivity, specificity, PPV, odds ratio, PPR and PAR (Supplementary Table 2). Line graphs show mean quantitative burden stratified by classification group and dementia status. Colours correspond to thresholds or staging criteria as shown in the figure key. Analyses were performed using averaged pathology burden across frontal cortices, hippocampal subfields (CA1–CA4), subiculum/parasubiculum, entorhinal–transentorhinal and fusiform cortices. Full statistical outputs are provided in the Supplementary material. *Bottom*: Circular ring plots show the frequency and co-occurrence of neuropathological and clinical features across controls, PD without dementia and combined PDD/DLB. Segments indicate the proportion of cases with high Lewy body burden (≥75th percentile) in frontal and medial temporal regions, intermediate-to-high ADNC, orthostatic hypotension, ischaemic pathology and dementia (where relevant). Overlapping segments illustrate co-occurrence within individuals. Colours correspond to the feature key shown beneath the plots. DLB = dementia with Lewy bodies; LB = Lewy bodies; LBD = Lewy body diseases; OR = odds ratio; PAR = pathology absence ratio; PD = Parkinson’s disease; PDD = Parkinson’s disease dementia; PPV = positive predictive value. Created in BioRender. Jaunmuktane, Z. (2026) https://BioRender.com/hpw73ng.

### Quantitative digital neuropathology

For quantitative image analysis, regions of interest (ROIs) (Supplementary Table 1) were annotated manually on NZConnect (web-based) or NDP.view2 (offline) (Hamamatsu) digital whole slide image viewing platform, according to predefined neuroanatomical landmarks, as detailed in the Supplementary material. In each brain region, a small circular annotation, named ‘! BACKGROUND’ with 10.5 µm diameter was positioned where background staining was visually most intense. The annotations were downloaded as NDPA files using a custom Python script and imported into QuPath v.0.5.1^31^ using a custom Groovy script for image analysis. To address staining variability across archival slides, colour deconvolution was performed on all analysed slides using stain vectors derived from a large composite of 270 whole slide images spanning the cohort, and the mean deconvoluted 3,3′-diaminobenzidine (DAB) intensity within each background annotation was used to generate region-specific, background-corrected thresholds for automatic Triangle^32^ (Aβ) or Otsu^33^ (pTau) detection, or as input to the StarDist model^34^ for Lewy body segmentation, resulting in %Aβ, %pTau or LB/mm^2^ readouts per ROI (Supplementary Figs 7–10). Detailed image analysis protocols are described in the Supplementary material.

### Semi-quantitative assessment of misfolded protein pathology

Semi-quantitative scoring was used to validate the quantitative image-analysis pipeline. α-Synuclein, Aβ and pTau burden were assessed semi-quantitatively by one author (N.C.), blinded to clinical and pathological diagnosis. Scoring was performed at ×40 magnification on digitized whole slide images across all ROIs on a 21-inch monitor with 2560 × 1440 pixel resolution, using a five-tier scale for α-synuclein (0 = none, 1 = rare, 2 = occasional, 3 = moderately frequent, 4 = frequent) and a four-tier scale for Aβ and pTau (0 = none, 1 = occasional, 2 = moderately frequent, 3 = frequent), shown in Fig. 1.

### Pathology burden classification and diagnostic performance metrics

The quantitative burden of α-synuclein, Aβ and pTau in the frontal cortex and hippocampal regions was averaged and binarized into ‘high’ and ‘low’ groups, with thresholds set at the 50th (median), 65th, 75th, 85th and 90th percentiles across all cases to assess the association with dementia. In parallel, established neuropathological criteria were used for binary classification: cortical Lewy body presence corresponding to Braak stages 5–6, Aβ corresponding to Thal phases 4–5, pTau corresponding to Braak and Braak stages IV–VI and intermediate-to-high ADNC (Supplementary material, data for Fig. 1).

To evaluate how well each pathological classification distinguished dementia status, we calculated sensitivity, specificity, odds ratio (OR), positive predictive value (PPV), pathology absence ratio (PAR) and pathology presence ratio (PPR)^35,36^ (Fig. 1, Supplementary material, data for Table 1 and Supplementary Table 2). For cross-group comparability, values for OR, PAR and PPR were normalized by dividing each by the maximum observed value across all groups for that marker.^37^ These metrics were used to generate radar plots (Fig. 1, middle).

### Logistic regression modelling of dementia risk

To evaluate the relationship between predictors and dementia status, logistic regression models were fitted using binomial family generalized linear models (glm).^38^ Binary logistic regression assessed the effect of ischaemic pathology on dementia risk among individuals with OH, stratified by combined misfolded protein burden. Multivariable logistic regression models stratified by *APOE* genotype included continuous predictors: α-synuclein (LB/mm^2^), Aβ (%Aβ) and pTau (%pTau) entered as linear terms, and categorical predictors: sex, ischaemic pathology, disease duration (<15 versus ≥15 years) and age at death (<77 versus ≥77 years), entered as binary factors; with cut-offs corresponding to cohort medians. Predicted dementia probabilities were visualized using the sjPlot::plot_model() function in R^39^ or continuous predictors. Marginal effects plots show predicted probability of dementia across the observed range with 95% confidence intervals (CI), and for categorical variables group-wise predicted probabilities with Wald test *P*-values are shown. For each *APOE* group, predicted probabilities were computed from the fitted multivariable models.


$$\begin{array}{rl} & full\;model<-glm(Dementia∼\alpha -synuclein\;burden+amyloid-\beta \;burden\\ & \;\;\;\;\;\;\;\;\;\;\;\;\;\;\;+pTau\;burden+ischaemia+biological\;sex+disease\;duration\\ & \;\;\;\;\;\;\;\;\;\;\;\;\;\;\;\;\;\;+age\;at\;death,data=dataset,family=binomial) \end{array}$$

Cumulative risk heatmap and decision-tree predictions are described in the Supplementary material.

### Quantitative regional pathology-driven disease progression modelling

To identify distinct patterns of pathology progression, we applied the SuStaIn algorithm^40^ to three *z*-score normalized datasets: (i) quantitative and (ii) semiquantitative regional α-synuclein pathology across 11 brain regions; and (iii) combined quantitative α-synuclein, Aβ and pTau burden data from cortical and medial temporal regions, excluding the amygdala. We generated a synthetic low-burden control set (50 cases with 0%–5% burden across regions) to provide a stable reference distribution for *z*-score normalization, thereby avoiding confounds from incidental Lewy and AD-type pathology in non-neurodegenerative controls. Synthetic controls were used only for standardization and SuStaIn modelling; all subsequent analyses of subtype characteristics were based on the LBD cases. Exact SuStaIn parameters and analysed regions are described in the Supplementary material.

Sankey diagrams were generated to illustrate the distribution of sex, *APOE* genotype, OH, ischaemic pathology, ADNC and dementia diagnosis across SuStaIn-derived subtypes.

### Statistical analysis

Normality of quantitative pathology measures (LB/mm^2^, Aβ % area and pTau % area) was assessed using Shapiro–Wilk tests; all variables were significantly non-normal (*P* < 0.05; Supplementary material), supporting the use of non-parametric analyses. Group differences were therefore evaluated using Kruskal–Wallis tests with Dunn’s Benjamini–Hochberg-corrected *post hoc* comparisons (Figs 1, 2A and B and Supplementary Figs 1, 4 and 6) or Mann–Whitney U-tests (Fig. 3 and Supplementary Fig. 2), as appropriate. Because no suitable non-parametric ANCOVA analogue exists for skewed continuous and ordinal measures, covariates were not included in group comparisons. Associations between semi-quantitative and quantitative pathology measures were assessed using Spearman’s rank correlation (Fig. 1), and distributional differences between *APOE* genotypes using two-sided Kolmogorov–Smirnov tests (Fig. 2C). Differences in categorical variables, including dementia status across staging schemes and pathology thresholds, were evaluated using χ^2^ or Fisher’s exact tests (Fig. 2D, Table 1 and Supplementary Tables 3–5). Dementia risk modelling was performed using univariate binary logistic regression (Fig. 4B) and multivariate logistic regression within *APOE* ɛ3 and ɛ4 subgroups (Fig. 5A), with significance assessed using Wald tests. Full methodological details are provided in the Supplementary material. All statistical analyses were conducted in R (v.4.4.2, RStudio v.2024.12.0.467),^41^ except for SuStaIn staging and downstream subtype analysis, which were performed in Python (Python v.3.9.21,^42^ Jupyter Notebook v.7.3.2).^43^ A large language model (ChatGPT, OpenAI) was used to assist in generating R code for statistical analyses and data visualization. Full statistical outputs and test specifications are provided in the Supplementary material for each figure and table. All code, statistical tests and outputs were reviewed manually and validated by the authors to ensure methodological appropriateness and accuracy of results.

**Table 1:** Clinical, demographic and neuropathological characteristics of study cohorts

Cohort	*n*	Male, *n* (%)	Age at onset, years	Disease duration, years	Age at death, years	*APOE* ɛ4 carriers, *n* (%)	Dementia, *n* (%)	Orthostatic hypotension, *n* (%)	Ischaemic pathology, *n* (%)
PD	130	**72/130 (55%)^a^**	60.7 ± 11.7	**15.9 ± 8.6^a^**	**76.3 ± 8.4^a,b^**	**26/110 (24%)^a^**	**0/130 (0%)^a,d^**	**39/59 (66%)^b^**	35/130 (27%)
PDD	146	**91/146 (62%)^b^**	59.8 ± 12.2	**17.7 ± 9.7^a^**	**77.4 ± 7.5^a,b^**	31/119 (26%)	**146/146 (100%)^b^**	**55/76 (72%)^b^**	51/146 (35%)
DLB	24	**20/24 (83%)^b,c^**	64.0 ± 7.3	**8.4 ± 3.2^c,e^**	**72.3 ± 6.9^b,c,e^**	**11/20 (55%)^b,c^**	**24/24 (100%)^b^**	**7/8 (88%)^b^**	6/24 (25%)
PDD + DLB	170	**111/170 (65%)^b^**	60.4 ± 11.7	16.4 ± 9.6	**76.7 ± 7.6^b^**	42/139 (30%)	**170/170 (100%)^b^**	**62/84 (74%)^b^**	57/170 (34%)
Controls	99	**43/99 (43%) ^a,d,e^**	–	–	**83.6 ± 10.8^a,c,d,e^**	**10/56 (18%)^a^**	**0/99 (0%)^a,c,d^**	**0/14 (0%)^a,c,d,e^**	33/99 (33%)
**Cohort**	***n***	**Lewy body (LB/mm^2^)**	**%pTau**	**%Aβ**	**α-Synuclein Braak stage**	**Aβ Thal phase**	**pTau Braak and Braak stage**	**ADNC**	**TDP-43 LATE stage**
PD	130	**1.147 [0.011–21.779]^a,b,e^**	**0.278 [0.005–7.23]^a,b,e^**	**0.054 [0.002–5.054]^a,b,e^**	**6 [0–6]^a,b,e^**	**1 [0–5]^a,b,e^**	**2 [0–4]^a,b,e^**	**1 [0–2]^a,b,e^**	9/79 (11%)
PDD	146	**3.922 [0.034–34.998]^a,b,c^**	**0.65 [0.007–18.803]^c^**	**0.453 [0.001–7.846]^a,c^**	**6 [0–6]^b,c^**	**3 [0–5]^a,c^**	**2 [0–6]^a,c^**	**1 [0–3]^a,c^**	18/107 (17%)
DLB	24	**7.372 [0.144–47.453]^b,c^**	**1.611 [0.056–19.313]^c^**	**1.68 [0.011–10.623]^b,c,e^**	**6 [4–6]^b,c^**	**4 [0–5]^b,c,e^**	**3 [1–6]^b,c,e^**	**2 [0–3]^b,c,e^**	3/14 (21%)
PDD + DLB	170	**4.261 [0.034–47.453]^b,c^**	**0.715 [0.007–19.313]^c^**	**0.664 [0.001–10.623]^c^**	**6 [0–6]^b,c^**	**3 [0–5]^c^**	**2 [0–6]^c^**	**1 [0–3]^c^**	21/121 (17%)
Controls	99	**0.026 [0–6.265]^a,c,d,e^**	**0.632 [0.003–11.015]^c^**	**0.446 [0.002–11.205]^a,c^**	**0 [0–6]^a,c,d,e^**	**2 [0–5]^a,c^**	**2 [0–6]^a,c^**	**1 [0–3]^a,c^**	4/66 (6%)

ADNC = Alzheimer's disease neuropathological change; DLB = Dementia with Lewy bodies; LATE = Limbic-predominant age-related TDP-43 encephalopathy; PD = Parkinson's disease; PDD = Parkinson's disease with dementia. Values are the mean ± standard deviation for age at onset, disease duration and age at death. Age at onset was calculated from reported symptom onset. Disease duration is symptom onset to death. Lewy body density, percentage immunoreactivity of amyloid-β (%Aβ) and percentage immunoreactivity of phosphorylated Tau (%pTau) are quantitative averages across defined cortical and medial temporal regions. Values for Lewy body density, %Aβ and %pTau, α-synuclein Braak stage, Aβ Thal phase, pTau Braak & Braak stage and Alzheimer’s disease neuropathological change (ADNC) level as per National Institute on Aging-Alzheimer’s Association (NIA-AA) are the median with [range]. ADNC: 0–3; Braak stage: 0–6; Aβ Thal: 0–5; pTau Braak & Braak stage: 0–VI (numbered 0–6). TDP-43 LATE staging is represented as percentage of cases, because all positive cases correspond to stage 2. ‘–’ indicates not applicable. *P*-values correspond to Benjamini–Hochberg-adjusted values using Fisher’s exact tests, χ^2^ proportion tests or pairwise Mann–Whitney U-tests, as appropriate. Full statistics are provided in the Supplementary material, statistics tables.

Bold text indicates significant pairwise differences and corresponds to the following significant contrasts:

^a^ *P* ≤ 0.05 versus DLB.

^b^ *P* ≤ 0.05 versus Controls.

^c^ *P* ≤ 0.05 versus PD.

^d^ *P* ≤ 0.05 versus combined PDD + DLB.

^e^ *P* ≤ 0.05 versus PDD.

**Figure 2:**
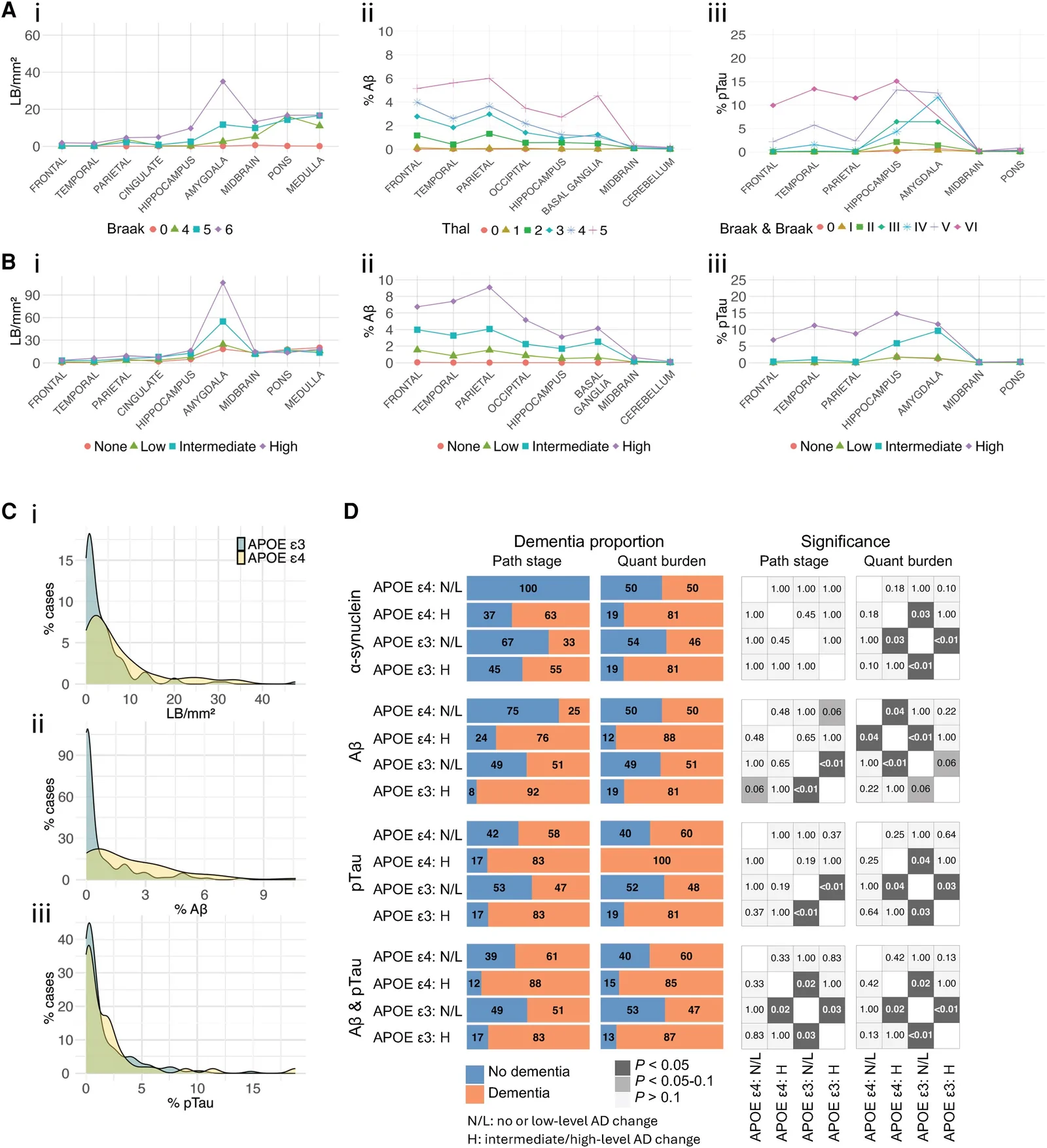
**Regional quantitative pathology burden stratified by traditional staging.** (**A**) Quantitative pathology burden across brain regions stratified by stage or phase of the corresponding misfolded proteinopathy. Quantitative pathology burden (*y*-axis) of Lewy bodies [**A**(**i**), LB/mm^2^], percentage area of Aβ [**A**(**ii**), % Aβ] and percentage area of phosphorylated tau [**A**(**iii**), %pTau] across brain regions (*x*-axis), stratified by stage or phase of the respective proteinopathy. [**A**(**i**)] Lewy body burden plotted by Braak stage (0, 4, 5, 6). [**A**(**ii**)] Aβ burden stratified by Thal phase (0–5). [**A**(**iii**)] pTau burden stratified by Braak and Braak stage (0–VI). Brain regions included are available in Supplementary material statistics data for Fig. 2B. Detailed statistical outputs are available in Supplementary material statistics data for Fig. 2A. Kruskal–Wallis tests with Dunn’s pairwise *post hoc* comparisons and Benjamini–Hochberg adjustment was performed per region for each proteinopathy. (**B**) Quantitative pathology burden across brain regions stratified by level of Alzheimer’s disease neuropathological change. Quantitative pathology burden (*y*-axis) of Lewy pathology [**B**(**i**), LB/mm^2^], percentage area of Aβ [**B**(**ii**), % Aβ] and percentage area of phosphorylated tau [**B**(**iii**), %pTau] across anatomical brain regions (*x*-axis), stratified by Alzheimer’s disease neuropathological change (ADNC) level (none, low, intermediate, high). Brain regions included are available in Supplementary material statistics data for Fig. 2B. Detailed statistical outputs are available in Supplementary material statistics data for Fig. 2B. Kruskal–Wallis tests with Dunn’s pairwise *post hoc* comparisons and Benjamini–Hochberg adjustment was performed per region for each proteinopathy. (**C**) Distribution of quantitative misfolded protein pathology burdens stratified by *APOE* genotype. Kernel density distributions plots show the percentage of cases (*y*-axis) across the range of measured pathology burden (*x*-axis) for Lewy bodies [**C**(**i**), LB/mm^2^], percentage area of Aβ [**C**(**ii**), %Aβ] and percentage area of phosphorylated tau [**C**(**iii**), %pTau], stratified by *APOE* genotype. Blue represents *APOE* ɛ3 carriers; yellow represents *APOE* ɛ4 carriers. These plots visualize the frequency distribution of pathology burden across cases, highlighting variation in burden profiles by genotype. Exact two-sample Kolmogorov–Smirnov test performed for *APOE* ɛ3 versus ɛ4 carriers for each proteinopathy (α-synuclein *P* = 0.02, Aβ *P* = 9.7 × 10^−7^, pTau *P* = 0.52). (**D**) Comparison of pathology staging versus quantitative burden in predicting dementia frequency across *APOE* genotypes divided by severity of Alzheimer’s disease neuropathological change. *Left*: Bar plots show the proportion of LBD patients with (orange) and without (blue) dementia stratified by *APOE* genotype (ɛ3, ɛ4) and ADNC (NL = none/low; H = intermediate/high). For each pathology (α-synuclein, Aβ, pTau and combined Aβ + pTau), dementia frequency is compared using traditional staging (α-synuclein: Braak ≥5; Aβ: Thal ≥4; pTau: Braak and Braak ≥ IV; combined: intermediate/high ADNC) and quantitative burden thresholds (α-synuclein ≥75th percentile; Aβ ≥ 75th percentile; pTau ≥85th percentile). *Right*: Heat maps show the statistical significance of differences in dementia frequency between *APOE*/ADNC groups for each classification approach, assessed using χ^2^ tests or Fisher’s exact tests (for counts <5) with Bonferroni correction. The *P*-values are visualized by shading: *P* < 0.05, black; 0.05–0.1, dark grey; >0.1, light grey. In **C** and **D**, significant quantitative pathology burden was defined as ≥75th percentile for α-synuclein (LB/mm^2^) and Aβ (% area), and ≥85th percentile for pTau (% area). Quantitative burden was averaged across the following regions: superior and middle frontal gyri, CA1–CA4, subiculum, parasubiculum, entorhinal cortex, transentorhinal cortex and fusiform gyrus. Detailed statistical outputs are provided in the Supplementary material for Fig. 2D. AD = Alzheimer's disease; LB = Lewy bodies.

**Figure 3:**
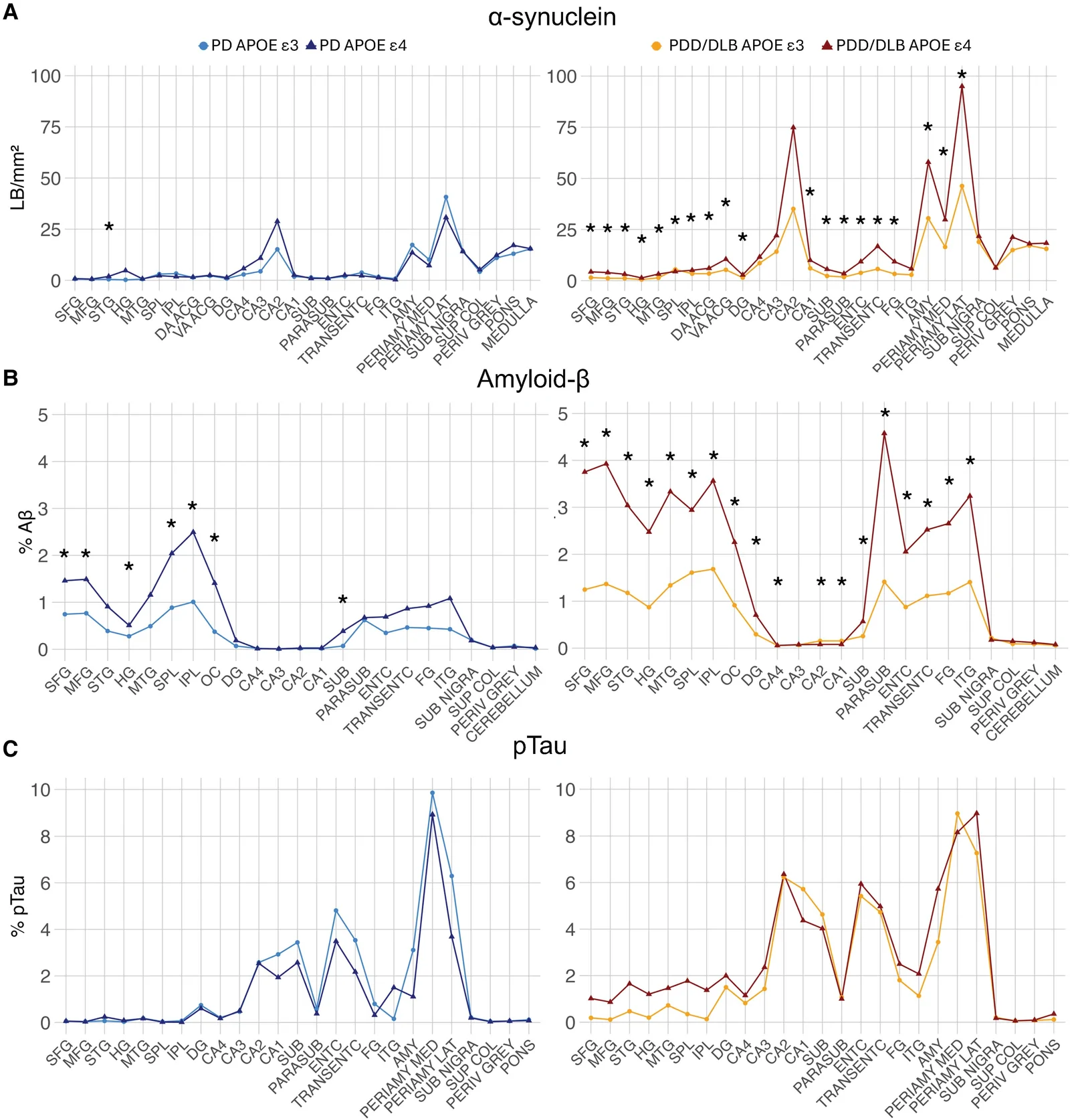
**Influence of *APOE* genotype on regional α-synuclein, Aβ and pTau pathology in Lewy body disease with and without dementia.** Lewy body (LB/mm^2^; **A**), percentage area of Aβ (%Aβ; **B**) and percentage area of phosphorylated tau (%pTau; **C**) quantitative pathology burden across multiple brain regions in Parkinson’s disease (PD) and dementia groups: Parkinson’s disease with dementia (PDD) and dementia with Lewy bodies (DLB), comparing *APOE* ɛ3 (light blue in PD, yellow in PDD/DLB) and *APOE* ɛ4 (dark blue in PD, brown in PDD/DLB) carriers. Lines indicate group mean pathology burden across regions; asterisks indicate statistically significant differences between genotype groups in each region (*P* < 0.05; Mann–Whitney U-test with Benjamini–Hochberg correction per region for each proteinopathy). AMY = amygdala; AMY_LS = amygdala lateral; AMY_MI = amygdala medial; CC = cerebellar cortex; DA ACG = dorso-anterior cingulate cortex; DG = dentate gyrus; ENTC = entorhinal cortex; FG = fusiform gyrus; HG = Heschl's gyrus; IPL= inferior parietal lobule; ITG = inferior temporal gyrus; MEDULLA/MTEGM = medulla tegmentum; MFG = middle frontal gyrus; MTG = middle temporal gyrus; OC = occipital; PAM_CX = periamygdaloid cortex; PARASUB = parasubiculum; PERIV GREY = periventricular grey matter; PONS/PTEGM = pontine tegmentum; SFG = superior frontal gyrus; SPL = superior parietal lobule; STG = superior temporal gyrus; SUB = subiculum; SUB NIGRA = substantia nigra; SUP COL = superior colliculus; TRANSENTC = transentorhinal cortex; VA ACG = ventro-anterior cingulate cortex. Brain region information is detailed in Supplementary Table 1, and detailed statistical outputs are available in Supplementary material, statistics data for Fig. 3.

**Figure 4:**
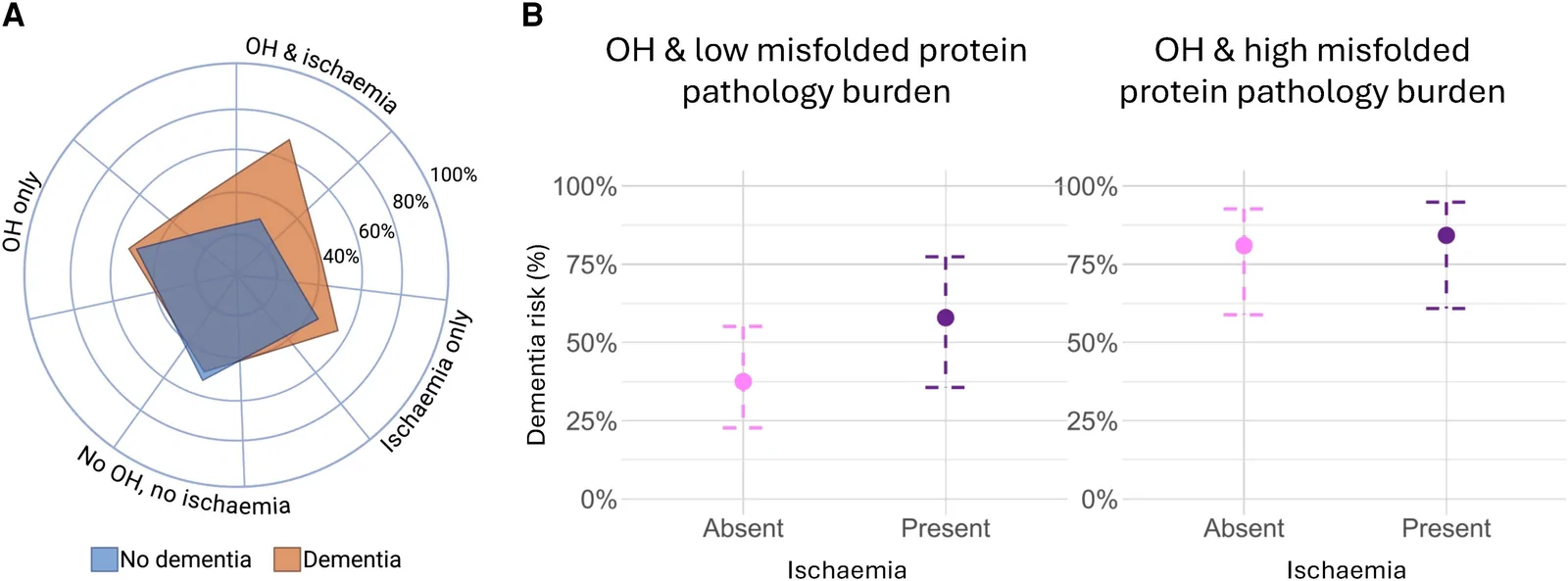
**Contribution of orthostatic hypotension and ischaemic pathology to dementia risk in Lewy body disease.** (**A**) Radar plot demonstrating the proportion of Lewy body disease (LBD) patients with (orange) and without (blue) dementia, stratified by the presence of orthostatic hypotension (OH), histological evidence of ischaemic brain pathology, both OH and ischaemic pathology, and neither. (**B**) Dot-and-whisker plots show dementia risk in patients with OH stratified by combined misfolded protein burden (low versus high, defined by composite quantitative α-synuclein, Aβ and pTau pathology) and presence or absence of histologically confirmed ischaemic pathology. In individuals with OH and low misfolded protein burden, ischaemic pathology is associated with a trend towards increased dementia risk, whereas in those with high misfolded protein burden, dementia risk is uniformly high regardless of ischaemic pathology; analysis performed with binary logistic regression. High versus low pathology burden was defined using the ≥75th percentile for α-synuclein and Aβ, and ≥85th percentile for pTau. Quantitative averages were calculated across the frontal cortex (superior and middle frontal gyri) and medial temporal regions, including hippocampal subfields (CA1–CA4, dentate gyrus, subiculum and parasubiculum), entorhinal and transentorhinal cortices and cortex of the fusiform gyrus.

**Figure 5:**
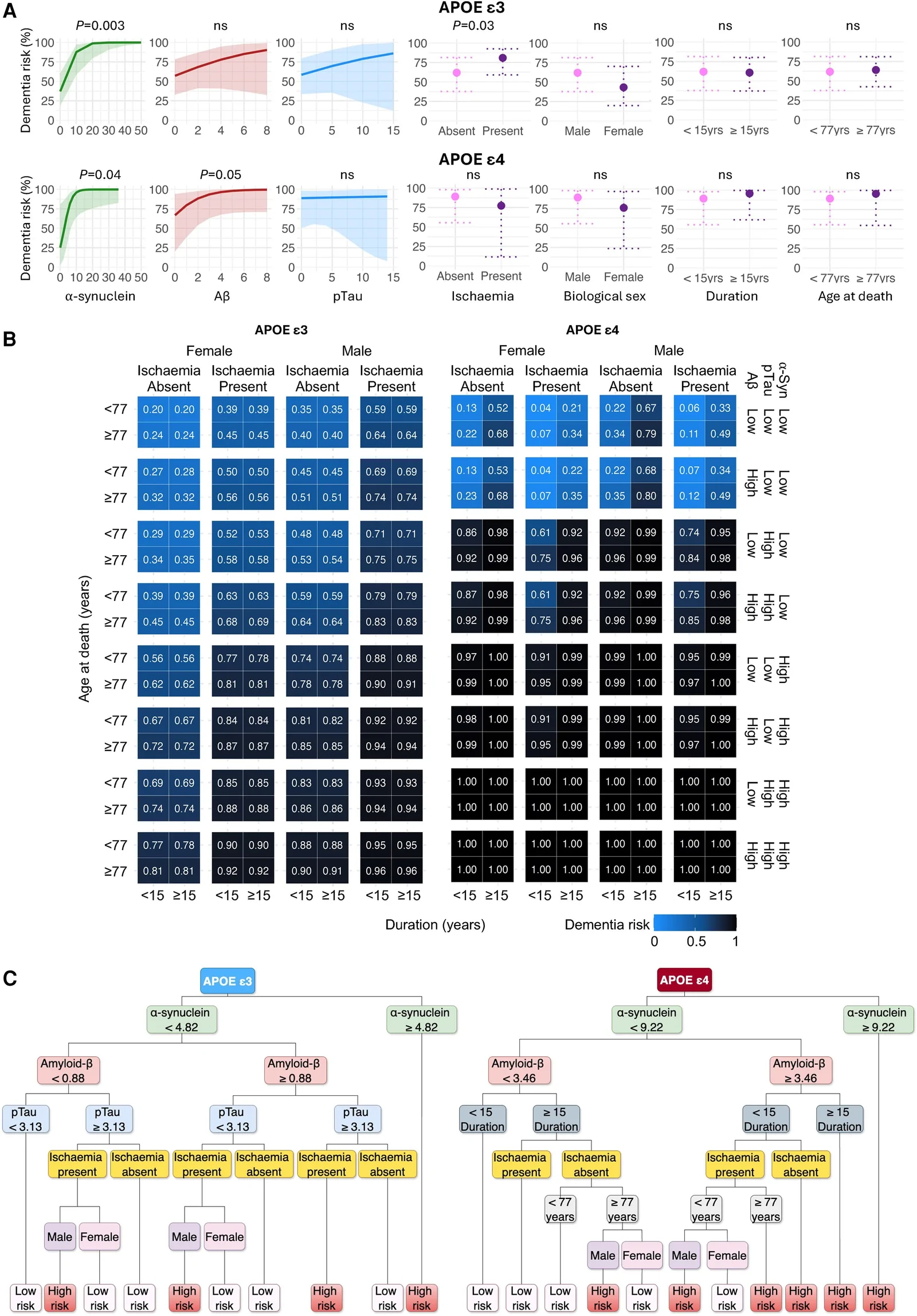
**Differential predictors of dementia risk in Lewy body disease: impact of *APOE* genotype, misfolded protein, ischaemic pathology, demographics and clinical features.** (**A**) Predicted dementia probabilities for individual variables from multivariate logistic regression models, stratified by *APOE* ɛ3 (top, *n* = 126) and ɛ4 (bottom, *n* = 46) genotype. *Left*: Three plots in each *row* display predicted probabilities as a function of increasing misfolded pathology burden. Dark lines and shaded bands indicate model-estimated means and 95% confidence intervals. *Right*: Four plots show predicted dementia probability across binary covariates. Large circles and dotted error bars indicate model-estimated group means and 95% confidence intervals. All effects were adjusted within a genotype-specific multivariate model using multivariate logistic regression with linear terms, and Wald tests were applied on model coefficients. Detailed statistical outputs are available in the Supplementary material statistics data for Fig. 5A. (**B**) Cumulative dementia risk heatmaps for *APOE* ɛ3 and ɛ4 genotypes, showing predicted probabilities of dementia [0–1 (absent–present), colour-coded from blue to black] for all combinations of clinical, demographic and dichotomized pathology burden variables. Significant pathology burden was defined using the ≥75th percentile for α-synuclein and Aβ, and ≥85th percentile for pTau. Quantitative averages were calculated across the frontal cortex (superior and middle frontal gyri) and medial temporal regions, including hippocampal subfields (CA1–CA4, dentate gyrus, subiculum and parasubiculum), entorhinal, transentorhinal and fusiform cortices. Detailed probability outputs are available in Supplementary material statistics data for Fig. 5B. (**C**) Decision trees illustrate hierarchical, threshold-based contributions of pathology and clinical variables to dementia risk in *APOE* ɛ3 (*left*) and ɛ4 (*right*) carriers. Each tree uses fixed, genotype-specific cut points for quantitative pathology measures (α-synuclein LB/mm^2^ and Aβ ≥ 75th percentile; pTau ≥85th percentile), applied consistently across all cross-validation folds. Additional splits incorporate binary ischaemic pathology (absent/present) and clinical variables (biological sex, age at death <77 versus ≥77 years, and disease duration <15 versus ≥15 years). Terminal nodes show the predicted probability of dementia. Models included quantitative α-synuclein, Aβ and pTau burden, ischaemic pathology, sex, age at death, and disease duration as input features. Analyses were performed in LBD cases with *APOE* ɛ3 (*n* = 181) and *APOE* ɛ4 (*n* = 69). ns = not significant. Created using draw.io (https://www.drawio.com).

## Results

### Demographic, clinicopathological and genetic characteristics of the cohort

A total of 399 cases were included in the study, comprising 99 controls without neurological disease and 300 LBD (130 PD, 146 PDD and 24 DLB; Fig. 1). Demographic, genetic and clinical characteristics are shown in Table 1 and Supplementary material. Across all disease groups, the proportion of males ranged from 55% to 83%, with no significant sex differences.

*APOE* ɛ4 carrier frequency was similar in PD (24%) and PDD (26%), but higher in DLB (55%) and lower in controls (18%), with overall group differences confirmed by χ^2^ testing (Table 1 and Supplementary material). Age at death and disease duration were comparable between PD (76.3 ± 8.4 and 15.9 ± 8.6 years, respectively) and PDD (77.4 ± 7.5 and 17.7 ± 9.7 years, respectively), but both were lower in DLB (72.3 ± 6.9 and 8.4 ± 3.2 years, respectively; non-parametric group comparisons in Supplementary material). OH was more frequent in PDD (72%) and DLB (88%) than in PD (66%), and ischaemic pathology was more common in PDD (35%) than in PD (27%) or DLB (25%) (Table 1; χ^2^/Fisher’s exact tests in the Supplementary material).

*APOE* ɛ3 carriers were older at death than ɛ4 carriers, regardless of dementia status, and disease duration was shorter in ɛ4 carriers with PD and PDD (Supplementary Tables 3 and 4 and Supplementary material). Semi-quantitative assessment showed limbic (stage 2) TDP-43 pathology in a small subset of cases (Table 1), correlating with increasing age at death but not with dementia status (Spearman correlation and χ^2^/Fisher’s exact tests; Supplementary Table 5 and Supplementary material). Given that TDP-43 pathology was not a frequent feature, automated quantification was not pursued.

### Quantitative pathology is correlated with semi-quantitative scores and enhances dementia prediction

Automated digital quantification of α-synuclein, Aβ and pTau showed strong correlations with semi-quantitative scores across frontal and hippocampal ROIs (Spearman’s ρ: α-synuclein = 0.72, Aβ = 0.80 and pTau = 0.76; all Spearman *P* < 0.0001), validating the digital quantitative pathology approach (Fig. 1, middle and Supplementary material).

Radar plots (Fig. 1, middle and Supplementary material) demonstrated that quantitative α-synuclein thresholds (particularly ≥75th percentile) outperformed Braak staging-based cortical Lewy pathology in dementia prediction, with higher specificity, OR and PPV. For Aβ (≥75th percentile) and pTau (≥85th percentile), quantitative thresholds performed similar to Thal, and Braak and Braak staging. Therefore, these thresholds were used to binarize regional quantitative pathology into ‘high’ and ‘low’ burden for all subsequent analyses (Fig. 1 and Supplementary material).

Quantitative pathology burden increased progressively across cortical and medial temporal regions with advancing pathological stage or phase for all three proteinopathies (Fig. 1 and Supplementary material). Quantitative burden measures (≥50th, 75th or 85th percentiles) consistently distinguished group differences more strongly than traditional staging, reflecting the added value of burden-based measurements.

In controls (Fig. 1, bottom), a minority of individuals showed either high Lewy pathology (2/99) or at least intermediate ADNC (30/99). Most PD patients without dementia, had low Lewy and ADNC pathology (83/130). A small subset had a high Lewy burden (10/130), intermediate-to-high ADNC (6/130), or both (1/130), despite preserved cognition. Among 170 LBD dementia cases, ore than half showed concurrent high Lewy pathology and/or ADNC (105/170). A minority had dementia despite low Lewy pathology and ADNC, absence of OH and no ischaemic pathology (8/170).

### Quantitative pathology burden intensifies with advancing disease stage and *APOE* ɛ4 genotype

Quantitative regional pathology measures aligned with established staging schemes and revealed additional gradations (Fig. 2, Supplementary Fig. 1 and Supplementary material). With advancing Lewy, Aβ and pTau stage/phase, pathology burden within already affected regions increased markedly, a nuance not captured by conventional staging, which documents only regional pathology presence rather than density. Lewy pathology showed the highest burden in limbic and neocortical regions at Braak stage 6; Aβ peaked in cortical and limbic regions at Thal phase 5; and pTau was greatest in medial temporal and neocortical regions at Braak and Braak stage VI (Fig. 2A). Quantitative burdens of all three proteinopathies also increased across ADNC levels, most prominently Lewy pathology in the amygdala and Aβ/pTau in limbic and neocortical regions, with Aβ additionally elevated in the basal ganglia (Fig. 2B).

In kernel density distributions of pathology burden, *APOE* ɛ4 carriers showed a broader, right-shifted distribution of Lewy body, Aβ and pTau burden compared with ɛ3 carriers, with significantly higher burdens for Lewy pathology and Aβ, but not pTau (two-sample Kolmogorov–Smirnov tests; Fig. 2C and Supplementary material).

Quantitative measures of Lewy, Aβ and pTau burden in frontal and medial temporal regions identified stronger group differences in dementia frequency than traditional staging approaches (Fig. 2D). Dementia prevalence was higher in *APOE* ɛ4 carriers and those with intermediate-to-high ADNC. Quantitative Lewy burden provided greater discrimination of dementia risk than Braak cortical stages 5–6, and quantitative Aβ and pTau measures outperformed Thal phases 4–5 and Braak stages V–VI, with similar discrimination when combined and compared with intermediate–high ADNC.

### *APOE* genotype modifies regional pathology burden in relationship to dementia status

In PD without dementia, Lewy pathology did not differ significantly by genotype except for higher burden in the superior temporal gyrus in ɛ4 carriers (*P* < 0.05). In PDD/DLB, ɛ4 carriers showed greater Lewy pathology across cortical and medial temporal regions, but not in the brainstem (Fig. 3A and Supplementary Fig. 2A). Aβ burden showed broader genotype effects: ɛ4 PD carriers had higher cortical and subicular Aβ even without dementia, with more widespread differences in PDD/DLB. Notably, ɛ4 carriers without dementia exhibited Aβ levels comparable to ɛ3 carriers with dementia (Fig. 3B and Supplementary Fig. 2B). pTau burden did not differ significantly by genotype, although region-specific patterns were observed in PDD/DLB, with higher cortical and limbic pTau in ɛ3 carriers and higher hippocampal pTau in ɛ4 carriers (Fig. 3C and Supplementary Fig. 2C).

### Orthostatic hypotension and ischaemic pathology elevate dementia risk at low misfolded protein burden

Given that OH is common in LBD and that reduced cerebral perfusion might contribute to ischaemia, we examined their overlap. Among dementia cases, 69% had both OH and ischaemic pathology versus 30% without dementia (Fig. 4A). The proportions with OH alone, ischaemic pathology alone, or neither were similar across groups. When stratified by combined misfolded protein burden, ischaemic pathology was linked to a non-significant trend towards higher dementia risk in low-burden cases (OR = 2.29, 95% CI = 0.72–7.30, *P* = 0.161), whereas in high-burden cases the dementia risk was elevated irrespective of ischaemic pathology (OR = 1.25, 95% CI = 0.24–6.50, *P* = 0.787) (Fig. 4B and Supplementary material).

### Multivariable modelling reveals distinct predictors of dementia

Multivariable logistic regression identified Lewy pathology as a significant predictor of dementia in both *APOE* ɛ3 (OR = 1.28, 95% CI 1.11–1.54; *P* = 0.003) and ɛ4 carriers (OR = 1.54, 95% CI 1.13–2.51; *P* = 0.04) (Fig. 5A). In ɛ3 carriers, ischaemic pathology was also independently associated with increased dementia risk (OR = 2.61, 95% CI 1.07–6.05; *P* = 0.035), whereas in ɛ4 carriers Aβ burden showed a borderline significant association (OR = 1.94, 95% CI 1.11–4.95; *P* = 0.05). Both ɛ3 and ɛ4 models demonstrated good overall performance (area under the curve = 0.77 and 0.92, respectively), with full calibration and goodness-of-fit statistics provided in the Supplementary material. Detailed regression coefficients, confidence intervals, Wald statistics and predicted-probability outputs are reported in the Supplementary material.

To visualize interactions among dementia prediction variables, we generated cumulative dementia-risk heat maps (Fig. 5B). In *APOE* ɛ4 carriers, predicted dementia probability remained high across nearly all combinations of pathology, vascular and demographic variables, even at low misfolded-protein burden, indicating a ceiling effect. In contrast, in ɛ3 carriers, non-proteinopathy factors, particularly ischaemia and male sex, produced marked increases in predicted dementia probability, indicating a stronger modulatory role for vascular and demographic influences in this genotype.

We also constructed rule-based surrogate decision-tree models using fixed, within-genotype thresholds for quantitative Lewy body, Aβ and pTau burden (Fig. 5C). Cross-validated performance was strong in both models (area under the curve = 0.879 for ɛ3; 0.923 for ɛ4), with corresponding optimal probability thresholds of 0.562 (ɛ3) and 0.583 (ɛ4), yielding sensitivities/specificities of 0.729/0.839 and 0.767/0.938, respectively. α-Synuclein burden emerged as the dominant predictor in both genotypes, with thresholds of 4.82 LB/mm^2^ (ɛ3) and 9.22 LB/mm^2^ (ɛ4). Aβ thresholds were lower in ɛ3 carriers (0.88% area) than in ɛ4 carriers (3.46% area). In ɛ3 carriers, pTau ranked third in importance, followed by ischaemia and sex; in ɛ4 carriers, disease duration ranked third, followed by ischaemia, age at death and sex.

### Data-driven modelling of Lewy pathology reveals four subtypes with clinical and genetic associations

SuStaIn applied to regional quantitative Lewy body burden in 295 LBD cases identified four progression subtypes (Fig. 6 and Supplementary Fig. 3). The amygdala subtype (*n* = 127, 43%) showed early amygdala involvement with concurrent brainstem pathology; the brainstem subtype (*n* = 94, 31.8%) showed early brainstem-restricted pathology; the cingulate subtype (*n* = 46, 15.6%) showed early anterior cingulate and concurrent brainstem involvement; and the neocortical subtype (*n* = 28, 9.5%) showed early frontal and temporal cortical involvement. These subtypes reflect distinct model-derived progression trajectories of regional Lewy body burden rather than cross-sectional statistical group differences. Notably, SuStaIn modelling of α-synuclein progression using semi-quantitative pathology scores identified progression patterns concordant with those derived from quantitative data-driven modelling, providing independent validation of the quantitative SuStaIn-derived subtypes (Supplementary Fig. 11). Differences were observed only in predicted subtype frequencies, probably reflecting limitations of semi-quantitative pathology scoring, which assigns maximal regional scores even when high pathological burden is confined to a small focus within ROIs.

**Figure 6:**
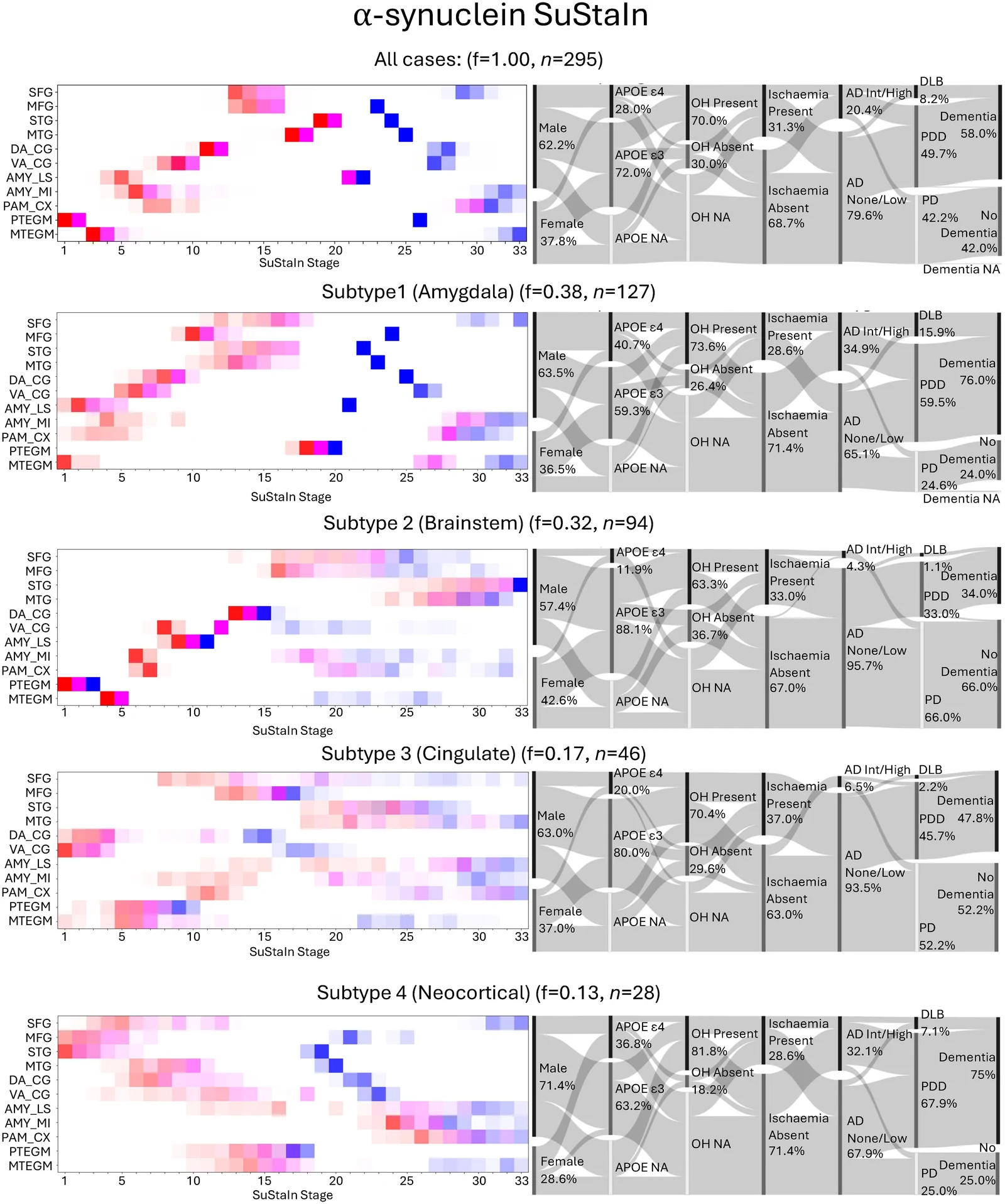
**Data-driven trajectories of Lewy pathology and associated clinical features in primary Lewy body disease.**  *Left*: Positional variance diagrams showing SuStaIn-inferred spatiotemporal trajectories of Lewy pathology (LB/mm^2^). The *x*-axis represents modelled disease stage; the *y*-axis lists the analysed regions (Supplementary Table 1). Colours denote abnormality relative to controls (mild = red, moderate = magenta, severe = blue), with colour intensity indicating certainty and pale shades reflecting higher positional variance. ‘f’ indicates the likelihood-weighted subtype frequency and ‘*n*’ the number of LBD cases assigned to each subtype. SuStaIn identified four trajectories: (1) amygdala (*n* = 127): early amygdala + brainstem involvement; (2) brainstem (*n* = 94): early brainstem-restricted pathology; (3) cingulate (*n* = 46): early anterior cingulate + brainstem involvement; and (4) neocortical (*n* = 28): early cortical involvement with delayed amygdala/brainstem involvement. *Right*: Sankey diagrams show the distribution of biological sex, *APOE* genotype (ɛ3/ɛ4), orthostatic hypotension (OH), ischaemic pathology, Alzheimer’s disease neuropathological change (ADNC) level, and dementia across quantitative pathology derived SuStaIn subtypes. Flow widths represent the proportion of cases per feature combination (excluding missing). Statistical comparisons (χ^2^, Bonferroni-corrected) are reported in Supplementary material statistics for Fig. 6.

Sankey analysis demonstrated marked genetic and co-pathology differences between quantitative pathology-derived SuStaIn subtypes, which were confirmed with pairwise χ^2^ tests (Bonferroni-corrected). The amygdala subtype showed the highest *APOE* ɛ4 frequency (40.7%) and AD co-pathology (34.9%), and the neocortical subtype also displayed elevated *APOE* ɛ4 (36.8%), substantial AD co-pathology (32.1%), and the highest rate of OH (81.8%). Both subtypes had the greatest dementia prevalence (76% and 75%, respectively) and the highest proportion of clinical DLB diagnosis (15.9% and 7.1%, respectively). In contrast, the brainstem subtype was enriched for *APOE* ɛ3 (88.1%), had minimal AD co-pathology (4.3%) and showed markedly lower dementia prevalence (34%), although dementia occurred in a subset without appreciable AD co-pathology. The cingulate subtype showed intermediate profiles across all variables. Statistically, the amygdala and brainstem subtypes differed most strongly, with significant differences in *APOE* genotype (adjusted *P* = 0.017), AD co-pathology (adjusted *P* < 0.0001), clinical diagnostic category (PD versus PDD versus DLB; adjusted *P* < 0.0001) and dementia prevalence (adjusted *P* < 0.0001). Additional significant contrasts included AD co-pathology between brainstem and neocortical subtypes (adjusted *P* = 0.0088) and between amygdala and cingulate subtypes (adjusted *P* = 0.0040); clinical diagnosis between brainstem and neocortical subtypes (adjusted *P* = 0.0138) and between amygdala and cingulate subtypes (adjusted *P* = 0.0321); and dementia prevalence between brainstem and neocortical subtypes (adjusted *P* = 0.0123).

Quantitative co-pathology further distinguished subtypes (Supplementary Fig. 4): the amygdala and neocortical subtypes showed higher cortical α-synuclein and greater limbic Aβ/pTau; the brainstem subtype showed minimal cortical Lewy body involvement and low Aβ/pTau; and the cingulate subtype displayed intermediate burden patterns. Overall, the amygdala and brainstem subtypes emerged as the two predominant and most biologically distinct trajectories, whereas the cingulate and neocortical subtypes captured less frequent variants of Lewy pathology progression.

### SuStaIn modelling of α-synuclein, Aβ and pTau identifies distinct *APOE* and co-pathology trajectories

SuStaIn applied to quantitative cortical and medial temporal (excluding amygdala) measures of α-synuclein, Aβ and pTau in 300 LBD cases inferred four data-driven progression subtypes, reflecting distinct predicted temporal trajectories of pathology aggregation rather than definite group differences (Fig. 7 and Supplementary Fig. 5). Subtype 1 (‘Aβ’, *n* = 133; 44.3%) showed early cortical Aβ accumulation, followed by cortical and then medial temporal α-synuclein. Subtype 2 (‘α-synuclein 1’, *n* = 83; 27.6%) resembled the classical Lewy trajectory, with medial temporal α-synuclein pathology preceding cortical involvement and later emergence of pTau aggregation. Subtype 3 (‘pTau’, *n* = 56; 18.6%) was defined by early medial temporal pTau followed by α-synuclein pathology appearance. Subtype 4 (‘α-synuclein 2’, *n* = 28; 9.3%) showed a neocortical Lewy body progression trajectory, beginning in the parietal cortex before medial temporal involvement.

**Figure 7:**
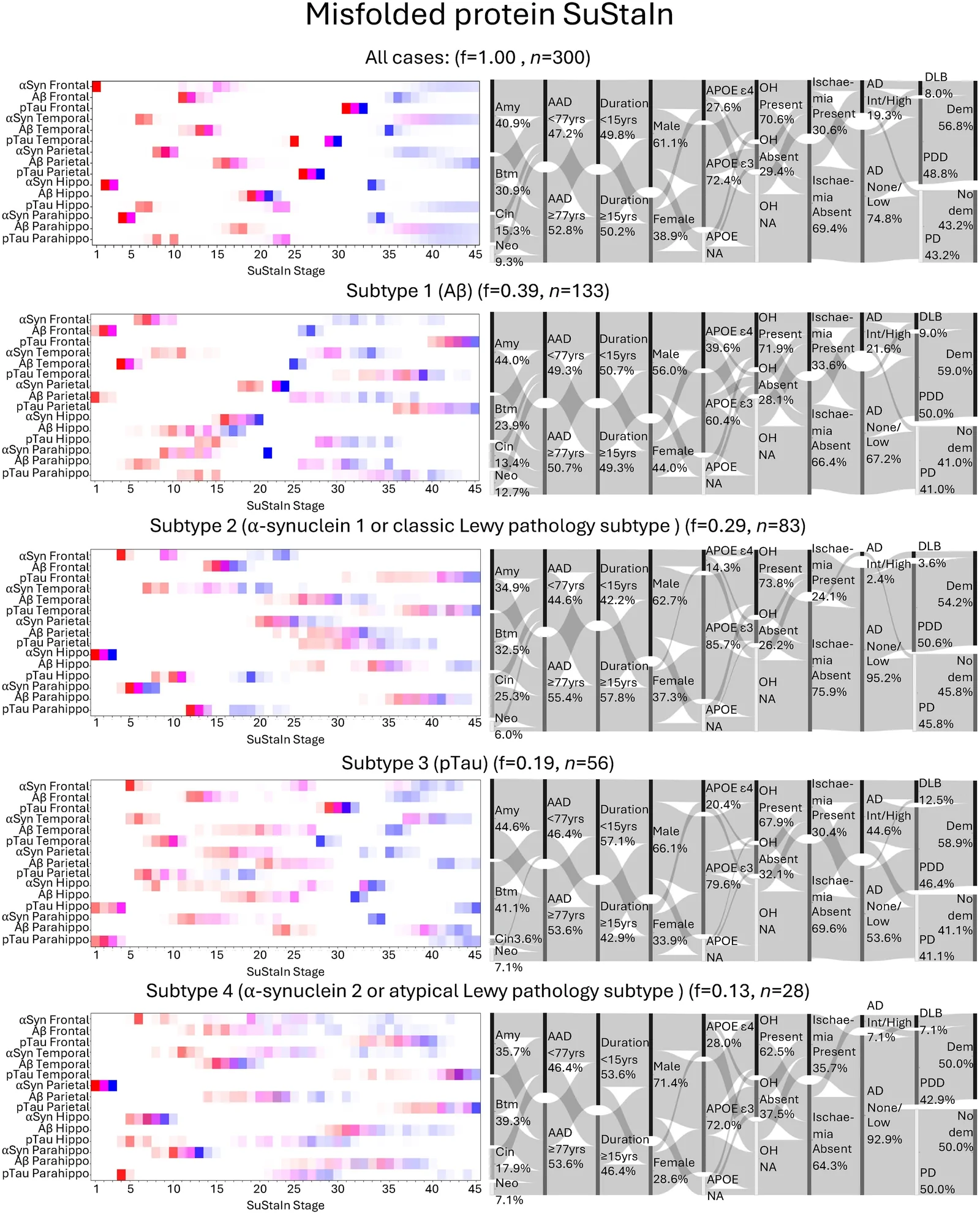
**Combined data-driven modelling of α-synuclein, Aβ and pTau pathology progression in primary Lewy body disease.**  *Left*: Positional variance diagrams showing SuStaIn-inferred spatiotemporal trajectories integrating α-synuclein, Aβ and pTau pathology. The *x*-axis represents disease stage; the *y*-axis lists the analysed regions [frontal, temporal and parietal cortices, hippocampus (dentate gyrus, CA4–CA1 and subiculum) and PHG/FG (parasubiculum, entorhinal/transentorhinal cortex and fusiform gyrus)]. Colours denote abnormality relative to controls (mild = red, moderate = magenta, severe = blue), with colour intensity indicating certainty and pale shading indicating greater positional variance. ‘f’ denotes the likelihood-weighted subtype frequency and ‘*n*’ the number of assigned cases. Modelling identified four progression subtypes: (i) Subtype 1 (Aβ-first; *n* = 133): early cortical Aβ; (ii) Subtype 2 (α-synuclein 1; *n* = 83): early medial temporal α-synuclein; (iii) Subtype 3 (pTau-first; *n* = 56): early medial temporal pTau; and (iv) Subtype 4 (α-synuclein 2; *n* = 28): early parietal α-synuclein. *Right*: Sankey diagrams show associations between the four subtypes and clinicopathological variables: α-synuclein SuStaIn-based subtypes [amygdala (Amy), brainstem (Btm), cingulate (Cin) and neocortical (Neo)], age at death (AAD, <77/≥77 years), disease duration (<15/≥15 years), sex, *APOE* genotype (ɛ3/ɛ4), orthostatic hypotension (OH), ischaemic pathology, Alzheimer’s disease neuropathological change (ADNC; None/Low versus Intermediate/High), and dementia status [dementia present (Dem) or no dementia (No dem)]. Flow widths represent the proportion of cases per feature combination (excluding missing). The χ^2^ results (Bonferroni-corrected) are provided in the Supplementary material, statistics for Fig. 7.

Sankey visualization showed clear genetic and co-pathology distinctions between SuStaIn subtypes, which were confirmed by pairwise χ^2^ tests (Bonferroni-corrected). The ‘Aβ’ subtype exhibited the highest *APOE* ɛ4 frequency (39.6%) and elevated intermediate-to-high ADNC (21.6%). The ‘α-synuclein 1’ subtype was enriched for *APOE* ɛ3 (85.7%) and showed the lowest ADNC burden (2.4%). The ‘pTau’ subtype had increased intermediate-to-high ADNC (44.6%) and moderate dementia prevalence (58.9%), whereas the ‘α-synuclein 2’ subtype showed minimal ADNC (7.1%). Other clinical variables, including dementia prevalence, ischaemic pathology, OH and age at death, did not differ significantly across subtypes. Statistical testing identified differences in *APOE* genotype (adjusted *P* = 0.05) and AD co-pathology (adjusted *P* = 0.00048) between the ‘Aβ’ and ‘α-synuclein 1’ subtypes, and in AD co-pathology between ‘α-synuclein 1’ and ‘pTau’ (adjusted *P* < 0.000001). No additional subtype comparisons remained significant after correction (Supplementary material for Fig. 7).

Quantitative burden measures further differentiated the subtypes (Supplementary Fig. 6). Lewy body load was highest in the ‘α-synuclein 1’ subtype, Aβ burden was dominant in the ‘Aβ’ subtype and elevated in the ‘pTau’ subtype, and pTau burden was selectively increased in the ‘pTau’ subtype, whereas the ‘α-synuclein 2’ subtype showed comparatively low to intermediate burdens of all three proteinopathies.

## Discussion

Through the implementation of quantitative pathology, this study advances the clinicopathological characterization of primary LBD by highlighting genotype-specific dementia risk and revealing novel primary LBD-specific progression patterns. Central to this study is the use of analytical approach suited to archival histological material, reflecting the practical realities of long-running brain-bank cohorts, where comprehensive re-staining is neither feasible nor representative of routine neuropathological practice.

### Quantitative pathology outperforms traditional staging in predicting dementia risk

The traditional Braak, Braak and Braak stage and Thal phase schemes summarize the regional extent of Lewy, AD-type pTau and Aβ pathology, respectively, whereas quantitative measures capture the severity within affected regions; in our analyses, quantified pathology was correlated more closely with dementia and enabled finer genotype–pathology comparisons. In this cohort, Lewy pathology progression to higher Braak stages reflected both regional spread and increasing burden, with particularly marked amygdala Lewy accumulation consistent with the influence of concomitant AD pathology. Quantitative cortical Lewy pathology burden provided stronger discrimination of dementia status than regional distribution alone, outperforming Braak staging.^25^ These findings are in line with previous reports, based on semi-quantitative assessments, showing that cortical Lewy burden is correlated more closely with cognition than regional extent.^4,44^ Together, these findings support incorporation of automated quantitative regional pathology burden to improve prediction of disease milestones and refine models of disease progression.

TDP-43 pathology was rare and correlated with age as reported in the general population,^45^ suggesting that it is not a major dementia risk in primary LBD.

### Male sex increases dementia risk in specific clinicopathological contexts

In our models, male sex increased dementia probability in both *APOE* genotypes, with a stronger effect in ɛ3 carriers in the presence of ischaemic pathology. Although *APOE* ɛ4 remained the main dementia risk factor, male sex further increased risk in ɛ4 carriers with longer disease duration and older age at death. These patterns are consistent with reports of greater vascular comorbidity in men with dementia, although other studies note faster age-related cognitive decline in females.^46,47^ Overall, these findings suggest that sex-specific dementia risk in LBD might depend on both genotype and co-pathology. Future studies should investigate vascular, hormonal and inflammatory mechanisms underlying these associations and test whether sex-stratified risk models improve dementia prediction.

### *APOE* ɛ4 impacts regional pathology burden and dementia risk in LBD

*APOE* ɛ4 carriers showed higher cortical and limbic but not brainstem Lewy pathology, suggesting that ɛ4 modulates progression to dementia rather than disease onset, and consistent with reports linking ɛ4 to greater cortical α-synuclein and Aβ burden and increased dementia risk in LBD.^8-10^ PD ɛ4 carriers without dementia had Lewy, Aβ and pTau burdens comparable to PDD ɛ3 carriers, consistent with the established role of ɛ4 in accelerating Aβ deposition in AD^48-50^ and suggesting a similar mechanism in LBD. This might involve impaired Aβ clearance, enhanced seeding and altered lipid metabolism that might facilitate Aβ and α-synuclein aggregation.^51-54^

Quantitative analyses indicate genotype-specific thresholds for dementia in LBD, with α-synuclein burden as the principal determinant, followed by Aβ, across *APOE* genotypes. *APOE* ɛ4 carriers with dementia had higher Lewy and Aβ burden than ɛ3 carriers. These findings suggest that ɛ4 promotes greater accumulation of pathology and modifies the threshold for dementia onset, with ɛ4 carriers tolerating higher α-synuclein and Aβ load before dementia onset. Longitudinal quantitative *in vivo* imaging with clinical and genotype correlation will be required to confirm this directly. The absence of independent predictive value for pTau indicates that it is largely downstream of Aβ, consistent with the amyloid cascade hypothesis.^55^

Taken together, these findings support a multifactorial role for *APOE* ɛ4 in both α-synuclein and Aβ accumulation, whereby enhanced amyloidogenic processes interact with α-synuclein to shape regional, particularly limbic, vulnerability and divergence in Lewy pathology progression patterns.

### Protein burden and *APOE* genotype modulate ischaemic effects in LBD

Previous work has linked OH to cognitive decline in PD, probably via hypoperfusion.^15,16^ In our cohort, OH frequency was similar in demented and non-demented LBD, but when combined with ischaemic pathology it amplified dementia risk in cases with low misfolded protein burden. At high burden, this effect disappeared, suggesting that proteinopathy overrides vascular modifiers.

### Data-driven modelling identifies divergent Lewy body progression patterns

Previous post-mortem and imaging studies have reported heterogeneity in Lewy pathology topography,^56-58^ motivating the use of data-driven modelling to capture biological variability in Lewy body progression. Using SuStaIn in a large primary LBD cohort, we observed the canonical caudal-to-rostral Lewy pathology progression pattern^25^ and identified additional trajectories that are broadly consistent with prior SuStaIn neuroimaging studies in PD^57^ and DLB^58^ and post-mortem study in a population cohort with Lewy pathology,^18^ while also revealing new insights.

Alongside the conventional brainstem-first trajectory, we identified a subtype with early amygdala involvement, similar to previous reports.^18,59^ Importantly, in our cohort, early amygdala involvement occurred in parallel with brainstem involvement, rather than representing an independent early initiation site. This contrasts with findings from population-based studies^18^  ^,59,60^ and observations in AD with amygdala-restricted Lewy pathology,^61^ suggesting selective vulnerability driven by local environment and *APOE* genotype. This subtype was enriched for *APOE* ɛ4 and advanced AD co-pathology, whereas the brainstem-first subtype was enriched for *APOE* ɛ3, and had lower AD co-pathology and dementia prevalence. Presence of both genotypes across subtypes, however, indicates that *APOE* modifies but does not uniquely determine propagation sequence.

We also identified two additional, less frequent trajectories, with one showing early anterior cingulate involvement occurring alongside brainstem pathology and the other with early isolated neocortical involvement. The neocortical subtype showed more frequent OH and, like the amygdala subtype, was enriched for *APOE* ɛ4 and advanced AD co-pathology. Both the neocortical and amygdala subtypes had higher dementia prevalence even without AD co-pathology, supporting findings that Lewy pathology burden is an independent dementia risk factor.^4,44^ These two trajectories were not observed in a recent SuStaIn study of a population cohort,^18^ suggesting that they might represent patterns specific to primary LBD.

Finally, multimodal SuStaIn modelling incorporating α-synuclein, Aβ and pTau indicated that some individuals follow Aβ-first or pTau-first trajectories, consistent with the notion that AD co-pathology might precede or accelerate α-synuclein aggregation in a subset of LBD cases. In contrast, the canonical Lewy-first trajectory, enriched for *APOE* ɛ3 and low AD burden, appears to be driven primarily by α-synuclein. Together, these results show that misfolded protein propagation in primary LBD is heterogeneous, *APOE* genotype dependent and influenced by AD co-pathology, underscoring the need for subtype-aware risk stratification and therapeutic trial design.

The main strength of this study is the validation and integration of automated whole slide image-based quantitative measurements of α-synuclein, Aβ and AD-type pTau pathology across multiple brain regions, linked to *APOE* genotype and clinical variables in a primary LBD cohort.

Our findings should be interpreted in the context of several limitations. Clinical information on OH and dementia might be incomplete owing to retrospective reporting, and *APOE* genotype data were unavailable for some cases. The DLB cases in our cohort had a younger age at death than typically reported in population-based studies,^62,63^ potentially reflecting the bias related to the limited sample size. The low prevalence of TDP-43 pathology reflects the nature of this cohort as a consecutive brain-donation series rather than a population-derived collection. Quantification of α-synuclein was restricted to Lewy bodies, because variable intensity of synaptic immunoreactivity in archival tissue precluded reliable detection of Lewy neurites. Lewy bodies were consistently detectable across cases, enabling implementation of a reproducible automated quantification pipeline suitable for long-term archival material. Neuritic and glial α-synuclein pathology were therefore not quantified. Comprehensive re-staining was not feasible given the scale and three-decade span of the collection and would not be expected entirely to overcome staining variability arising from historical differences in tissue fixation duration and fixative quality. Histological examination was restricted to one hemisphere, which might underestimate microscopic ischaemic burden if pathology was confined to the contralateral side. We also did not assess the extent or severity of large- and small-vessel disease. Furthermore, the absence of olfactory bulb tissue in our cohort precluded direct comparison with an earlier study identifying it as a frequent initial site of Lewy pathology in a population cohort.^18^ Likewise, the absence of peripheral autonomic tissues precluded comparison with a recent study that identified vagal and sympathetic body-first subtypes of LBD.^64^ Finally, modest sample sizes in genotype-stratified analyses and the cross-sectional design make it difficult to determine whether the observed associations are causal.

Despite these limitations, our study provides findings of translational relevance. The automated pipeline we developed for large-scale quantification of misfolded proteinopathies provides a foundation for standardizing digital pathology data across neurodegenerative diseases. By integrating *APOE* genotype, AD co-pathology, ischaemic pathology and clinical features, we identify distinct patterns of Lewy pathology propagation, underscoring the importance of multidimensional approaches to dementia risk modelling.

We also show that *APOE* ɛ3 and ɛ4 carriers differ in their pathological thresholds for dementia, indicating genotype-related differences in vulnerability.

These observations generate testable hypotheses regarding genotype-tailored therapeutic strategies and inform interpretation of threshold values in protein-based biomarkers, including seeding aggregation assays for fluid-based detection of α-synuclein, Aβ and pTau, in addition to PET imaging measures. Our findings might also motivate stratified genome-wide association study analyses within SuStaIn-defined subgroups to explore additional genetic contributions to dementia and other clinical milestones. Replication in independent brain-bank cohorts using comparable quantitative or semiquantitative pathology pipelines will be required to assess the generalizability of these findings.

## Conclusion

In conclusion, by integrating quantitative measures of misfolded protein burden with genotype and clinical data, we delineate biologically distinct but overlapping pathways to dementia in primary LBD. Together, these results extend existing models of disease progression and provide a quantitative, pathology-informed framework that might be useful for future therapeutic trial design.

## Supplementary Material

awag114_Supplementary_Data

## Data Availability

The raw quantitative pathological data, protocols, R and python code and key laboratory materials used and generated in this study are listed in a Key Resource Table (KRT) alongside their persistent identifiers at https://zenodo.org/records/18315069. Whole slide images of cases used in this study are available for viewing via UCL QSBB Digital Pathology Resource (RRID:SCR_025020). Anonymized clinical and pathology data are available upon reasonable request, if in compliance with regulatory ethical approval.

## References

[1] Emre M, Aarsland D, Brown R, et al Clinical diagnostic criteria for dementia associated with Parkinson’s disease. Mov Disord. 2007;22:1689–1707. 10.1002/mds.21507 17542011

[2] McKeith IG, Boeve BF, Dickson DW, et al Diagnosis and management of dementia with Lewy bodies: Fourth consensus report of the DLB Consortium. Neurology. 2017;89:88–100. 10.1212/WNL.0000000000004058 PMC5496518 28592453

[3] Braak H, Rüb U, Jansen Steur EN, Del Tredici K, de Vos RA. Cognitive status correlates with neuropathologic stage in Parkinson disease. Neurology. 2005;64:1404–1410. 10.1212/01.WNL.0000158422.41380.82 15851731

[4] Compta Y, Parkkinen L, O'Sullivan SS, et al Lewy- and Alzheimer-type pathologies in Parkinson’s disease dementia: Which is more important? Brain. 2011;134(Pt 5):1493–1505. 10.1093/brain/awr031 PMC4194668 21596773

[5] Jellinger KA, Korczyn AD. Are dementia with Lewy bodies and Parkinson’s disease dementia the same disease? BMC Med. 2018;16:34. 10.1186/s12916-018-1016-8 PMC5840831 29510692

[6] Guerreiro R, Ross OA, Kun-Rodrigues C, et al Investigating the genetic architecture of dementia with Lewy bodies: A two-stage genome-wide association study. Lancet Neurol. 2018;17:64–74. 10.1016/S1474-4422(17)30400-3 PMC5805394 29263008

[7] Kaivola K, Shah Z, Chia R, Scholz SW. Genetic evaluation of dementia with Lewy bodies implicates distinct disease subgroups. Brain. 2022;145:1757–1762. 10.1093/brain/awab402 PMC9423712 35381062

[8] Dickson DW, Heckman MG, Murray ME, et al *APOE* ɛ4 is associated with severity of Lewy body pathology independent of Alzheimer pathology. Neurology. 2018;91:e1182–e1195. 10.1212/WNL.0000000000006212 PMC6161556 30143564

[9] Tsuang D, Leverenz JB, Lopez OL, et al *APOE* ɛ4 increases risk for dementia in pure synucleinopathies. JAMA Neurol. 2013;70:223–228. 10.1001/jamaneurol.2013.600 PMC3580799 23407718

[10] Huang X, Chen P, Kaufer DI, Tröster AI, Poole C. Apolipoprotein E and dementia in Parkinson disease: A meta-analysis. Arch Neurol. 2006;63:189–193. 10.1001/archneur.63.2.189 16476806

[11] Masliah E, Rockenstein E, Veinbergs I, et al β-Amyloid peptides enhance α-synuclein accumulation and neuronal deficits in a transgenic mouse model linking Alzheimer’s disease and Parkinson’s disease. Proc Natl Acad Sci U S A. 2001;98:12245–12250. 10.1073/pnas.211412398 PMC59799 11572944

[12] Bassil F, Brown HJ, Pattabhiraman S, et al Amyloid-beta (Aβ) plaques promote seeding and spreading of alpha-synuclein and tau in a mouse model of Lewy body disorders with Aβ pathology. Neuron. 2020;105:260–275.e6. 10.1016/j.neuron.2019.10.010 PMC6981053 31759806

[13] Clinton LK, Blurton-Jones M, Myczek K, Trojanowski JQ, LaFerla FM. Synergistic interactions between Aβ, tau, and α-synuclein: Acceleration of neuropathology and cognitive decline. J Neurosci. 2010;30:7281–7289. 10.1523/JNEUROSCI.0490-10.2010 PMC3308018 20505094

[14] Hardy J . The relationship between Lewy body disease, Parkinson’s disease, and Alzheimer’s disease. Ann N Y Acad Sci. 2003;991:167–170. 10.1111/j.1749-6632.2003.tb07474.x 12846985

[15] Allcock LM, Kenny RA, Mosimann UP, et al Orthostatic hypotension in Parkinson’s disease: Association with cognitive decline? Int J Geriatr Psychiatry. 2006;21:778–783. 10.1002/gps.1562 16906622

[16] Ruiz Barrio I, Miki Y, Jaunmuktane ZT, Warner T, De Pablo-Fernandez E. Association between orthostatic hypotension and dementia in patients with Parkinson disease and multiple system atrophy. Neurology. 2023;100:e998–e1008. 10.1212/WNL.0000000000201659 PMC9990860 36526431

[17] De Pablo-Fernandez E, Tur C, Revesz T, Lees AJ, Holton JL, Warner TT. Association of autonomic dysfunction with disease progression and survival in Parkinson disease. JAMA Neurol. 2017;74:970–976. 10.1001/jamaneurol.2017.1125 PMC5710320 28655059

[18] Mastenbroek SE, Vogel JW, Collij LE, et al Disease progression modelling reveals heterogeneity in trajectories of Lewy-type α-synuclein pathology. Nat Commun. 2024;15:5133. 10.1038/s41467-024-49402-x PMC11180185 38879548

[19] Freeman R, Wieling W, Axelrod FB, et al Consensus statement on the definition of orthostatic hypotension, neurally mediated syncope and the postural tachycardia syndrome. Auton Neurosci. 2011;161:46–48. 10.1016/j.autneu.2011.02.004 21393070

[20] Bandres-Ciga S, Faghri F, Majounie E, et al NeuroBooster array: A genome-wide genotyping platform to study neurological disorders across diverse populations. Mov Disord. 2024;39:2039–2048. 10.1002/mds.29902 PMC11568947 39283294

[21] Chang CC, Chow CC, Tellier LC, Vattikuti S, Purcell SM, Lee JJ. Second-generation PLINK: Rising to the challenge of larger and richer datasets. GigaScience. 2015;4:7. 10.1186/s13742-015-0047-8 PMC4342193 25722852

[22] Taliun D, Harris DN, Kessler MD, et al Sequencing of 53,831 diverse genomes from the NHLBI TOPMed Program. Nature. 2021;590:290–299. 10.1038/s41586-021-03205-y PMC7875770 33568819

[23] Loh P-R, Danecek P, Palamara PF, et al Reference-based phasing using the Haplotype Reference Consortium panel. Nat Genet. 2016;48:1443–1448. 10.1038/ng.3679 PMC5096458 27694958

[24] Real R, Martinez-Carrasco A, Reynolds RH, et al Association between the *LRP1B* and *APOE* loci and the development of Parkinson’s disease dementia. Brain. 2023;146:1873–1887. 10.1093/brain/awac414 PMC10151192 36348503

[25] Braak H, Del Tredici K, Rub U, de Vos RA, Jansen Steur EN, Braak E. Staging of brain pathology related to sporadic Parkinson’s disease. Neurobiol Aging. 2003;24:197–211. 10.1016/s0197-4580(02)00065-9 12498954

[26] Thal DR, Rub U, Orantes M, Braak H. Phases of Aβ-deposition in the human brain and its relevance for the development of AD. Neurology. 2002;58:1791–1800. 10.1212/wnl.58.12.1791 12084879

[27] Braak H, Braak E. Neuropathological stageing of Alzheimer-related changes. Acta Neuropathol. 1991;82:239–259. 10.1007/BF00308809 1759558

[28] Hyman BT, Phelps CH, Beach TG, et al National Institute on Aging-Alzheimer’s Association guidelines for the neuropathologic assessment of Alzheimer’s disease. Alzheimers Dement. 2012;8:1–13. 10.1016/j.jalz.2011.10.007 PMC3266529 22265587

[29] Montine TJ, Phelps CH, Beach TG, et al National Institute on Aging-Alzheimer’s Association guidelines for the neuropathologic assessment of Alzheimer’s disease: A practical approach. Acta Neuropathol. 2012;123:1–11. 10.1007/s00401-011-0910-3 PMC3268003 22101365

[30] Nelson PT, Dickson DW, Trojanowski JQ, et al Limbic-predominant age-related TDP-43 encephalopathy (LATE): Consensus working group report. Brain. 2019;142:1503–1527. 10.1093/brain/awz099 PMC6536849 31039256

[31] Bankhead P, Loughrey MB, Fernandez JA, et al Qupath: Open source software for digital pathology image analysis. Sci Rep. 2017;7:16878. 10.1038/s41598-017-17204-5 PMC5715110 29203879

[32] Zack GW, Rogers WE, Latt SA. Automatic measurement of sister chromatid exchange frequency. J Histochem Cytochem. 1977;25:741–753. 10.1177/25.7.70454 70454

[33] Otsu N . A threshold selection method from gray-level histograms. IEEE Trans Syst Man Cybern. 1979;9:62–66.

[34] Schmidt U, Weigert M, Broaddus C, Myers G. Cell Detection with star-convex polygons. In: Frangi A, Schnabel J, Davatzikos C, Alberola-López C, Fichtinger G, eds. Medical image computing and computer assisted intervention – MICCAI 2018. MICCAI 2018. Lecture notes in computer science, Vol 11071. Springer; 2018.

[35] Altman DG, Bland JM. Statistics notes: Diagnostic tests 1: Sensitivity and specificity. BMJ. 1994;308:1552. 10.1136/bmj.308.6943.1552 PMC2540489 8019315

[36] Trevethan R . Sensitivity, specificity, and predictive values: Foundations, pliabilities, and pitfalls in research and practice. Front Public Health. 2017;5:307. 10.3389/fpubh.2017.00307 PMC5701930 29209603

[37] Jolliffe IT, Cadima J. Principal component analysis: A review and recent developments. Philos Trans A Math Phys Eng Sci. 2016;374:20150202. 10.1098/rsta.2015.0202 PMC4792409 26953178

[38] Dobson AJ, Barnett AG. An introduction to generalized linear models. 3rd ed. Chapman and Hall/CRC; 2008.

[39] Comprehensive R Archive Network (CRAN) . sjPlot: Data visualization for statistics in social science. Version R package; 2025. https://CRAN.R-project.org/package=sjPlot

[40] Young AL, Marinescu RV, Oxtoby NP, et al Uncovering the heterogeneity and temporal complexity of neurodegenerative diseases with Subtype and Stage Inference. Nat Commun. 2018;9:4273. 10.1038/s41467-018-05892-0 PMC6189176 30323170

[41] R Foundation for Statistical Computing . R: A language and environment for statistical computing; 2024. https://www.R-project.org/

[42] Python Software Foundation . Python: A dynamic, open source programming language; 2024. https://www.python.org/

[43] Kluyver T, Ragan-Kelley B, Pérez F, et al Jupyter Notebooks—A publishing format for reproducible computational workflows. In: Loizides F, Schmidt B, eds. Positioning and power in academic publishing: Players, agents and agendas. IOS Press; 2016:87–90.

[44] Mattila PM, Rinne JO, Helenius H, Dickson DW, Röyttä M. Alpha-synuclein-immunoreactive cortical Lewy bodies are associated with cognitive impairment in Parkinson's disease. Acta Neuropathol. 2000;100:285–290. 10.1007/s004019900168 10965798

[45] Nelson PT, Fardo DW, Wu X, Aung KZ, Cykowski MD, Katsumata Y. Limbic-predominant age-related TDP-43 encephalopathy (LATE-NC): Co-pathologies and genetic risk factors provide clues about pathogenesis. J Neuropathol Exp Neurol. 2024;83:396–415. 10.1093/jnen/nlae032 PMC11110076 38613823

[46] Kaur A, Fouad MH, Pozzebon C, Behlouli H, Rajah MN, Pilote L. Sex differences in the association between vascular risk factors and cognitive decline: A UK biobank study. JACC Adv. 2024;3:100930. 10.1016/j.jacadv.2024.100930 PMC11312777 39130034

[47] Anstey KJ, Peters R, Mortby ME, et al Association of sex differences in dementia risk factors with sex differences in memory decline in a population-based cohort spanning 20–76 years. Sci Rep. 2021;11:7710. 10.1038/s41598-021-86397-7 PMC8032756 33833259

[48] Hampel H, Hardy J, Blennow K, et al The amyloid-β pathway in Alzheimer’s disease. Mol Psychiatry. 2021;26:5481–5503. 10.1038/s41380-021-01249-0 PMC8758495 34456336

[49] Liu CC, Zhao N, Fu Y, et al ApoE4 accelerates early seeding of amyloid pathology. Neuron. 2017;96:1024–1032.e3. 10.1016/j.neuron.2017.11.013 PMC5948105 29216449

[50] Kim H, Kim S, Cho B, Shin J, Kim J. APOE ɛ4-dependent effects on the early amyloid pathology in induced neurons of patients with Alzheimer’s disease. Transl Neurodegener. 2022;11:45. 10.1186/s40035-022-00319-9 PMC9594913 36284363

[51] Liu CC, Liu CC, Kanekiyo T, Xu H, Bu G. Apolipoprotein E and Alzheimer disease: Risk, mechanisms and therapy. Nat Rev Neurol. 2013;9:106–118. 10.1038/nrneurol.2012.263 PMC3726719 23296339

[52] Verghese PB, Castellano JM, Holtzman DM. Apolipoprotein E in Alzheimer’s disease and other neurological disorders. Lancet Neurol. 2011;10:241–252. 10.1016/S1474-4422(10)70325-2 PMC3132088 21349439

[53] Zhao N, Attrebi ON, Ren Y, et al APOE4 exacerbates α-synuclein pathology and related toxicity independent of amyloid. Sci Transl Med. 2020;12:eaay1809. 10.1126/scitranslmed.aay1809 PMC8309690 32024798

[54] Jin Y, Li F, Sonoustoun B, et al APOE4 exacerbates α-synuclein seeding activity and contributes to neurotoxicity in Alzheimer’s disease with Lewy body pathology. Acta Neuropathol. 2022;143:641–662. 10.1007/s00401-022-02421-8 PMC9107450 35471463

[55] Hardy J, Selkoe DJ. The amyloid hypothesis of Alzheimer’s disease: Progress and problems on the road to therapeutics. Science. 2002;297:353–356. 10.1126/science.1072994 12130773

[56] Colloby SJ, McAleese KE, Walker L, et al Patterns of tau, amyloid and synuclein pathology in ageing, Alzheimer’s disease and synucleinopathies. Brain. 2025;148:1562–1576. 10.1093/brain/awae372 PMC12073977 39531734

[57] Zhou C, Wang L, Cheng W, et al Two distinct trajectories of clinical and neurodegeneration events in Parkinson’s disease. NPJ Parkinsons Dis. 2023;9:111. 10.1038/s41531-023-00556-3 PMC10344958 37443179

[58] Inguanzo A, Poulakis K, Mohanty R, et al MRI data-driven clustering reveals different subtypes of dementia with Lewy bodies. NPJ Parkinsons Dis. 2023;9:5. 10.1038/s41531-023-00448-6 PMC9859778 36670121

[59] Raunio A, Kaivola K, Tuimala J, et al Lewy-related pathology exhibits two anatomically and genetically distinct progression patterns: A population-based study of Finns aged 85+. Acta Neuropathol. 2019;138:771–782. 10.1007/s00401-019-02071-3 PMC6800868 31494694

[60] Raunio A, Myllykangas L, Kero M, Polvikoski T, Paetau A, Oinas M. Amygdala α-synuclein pathology in the population-based Vantaa 85+ study. J Alzheimers Dis. 2017;58:669–674. 10.3233/JAD-170104 PMC6218117 28482633

[61] Uchikado H, Lin WL, DeLucia MW, Dickson DW. Alzheimer disease with amygdala Lewy bodies: A distinct form of alpha-synucleinopathy. J Neuropathol Exp Neurol. 2006;65:685–697. 10.1097/01.jnen.0000225908.90052.07 PMC5706655 16825955

[62] Bentivenga GM, Baiardi S, Mastrangelo A, et al Clinical, neuropathological, and molecular characteristics of rapidly progressive dementia with Lewy bodies: A distinct clinicopathological entity? Alzheimers Res Ther. 2024;16:201. 10.1186/s13195-024-01565-x PMC11384710 39256877

[63] Sim J, Li H, Hameed S, Ting SKS. Clinical manifestations of early-onset dementia with Lewy bodies compared with late-onset dementia with Lewy bodies and early-onset Alzheimer disease. JAMA Neurol. 2022;79:702–709. 10.1001/jamaneurol.2022.1133 PMC9127709 35604656

[64] Andersen KB, Krishnamurthy A, Just MK, et al Sympathetic and parasympathetic subtypes of body-first Lewy body disease observed in postmortem tissue from prediagnostic individuals. Nat Neurosci. 2025;28:925–936. 10.1038/s41593-025-01910-9 PMC12081295 40082617

