# Supplementary material for: Quantitative pathology and APOE genotype reveal dementia risk and progression in Lewy body disease
Source: Brain. 2026 Mar 27;149(11):3905–22. doi: 10.1093/brain/awag114 (PMC13730591; doi:10.1093/brain/awag114)
Supplement: Supplementary file 1 [file awag114_supplementary_data.zip › brain-2025-02697-File010.pdf]

Quantitative pathology and APOE genotype reveal dementia risk and progression in Lewy body disease

Authors

Hemanth R Nelvagal[1,2,3], Nancy Chiraki[1,2,3], Toby Curless[3,4], Patrick W Cullinane[1,2,5,6], Alice Rockliffe[1,2], Samarth Pimparkar[1,2], Haruka Kawamura[2], Sophie Ollerenshaw[2], Isha Elahi[2], Sebastian Brandner[7,8], Lesley Wu[1], Raquel Real[1,3], Mina Ryten[3,9], John Hardy[3,7], Eduardo De Pablo Fernandez[1,2,5,10], Thomas T Warner[1,2,5,6], Huw R Morris[1,3,6], Yau Mun Lim[3,7], Zane Jaunmuktane[1,2,3,8]

Affiliations

1. Department of Clinical and Movement Neurosciences, UCL Queen Square Institute of Neurology, University College London, London, WC1N 3BG, United Kingdom
2. Queen Square Brain Bank for Neurological Disorders, UCL Queen Square Institute of Neurology, London, WC1N 1PJ, United Kingdom
3. Aligning Science Across Parkinson's (ASAP) Collaborative Research Network, Chevy Chase, MD 20815, USA
4. Department of Neuromuscular Diseases, UCL Queen Square Institute of Neurology, University College London, London, WC1N 3BG, United Kingdom
5. Reta Lila Weston Institute of Neurological Studies, UCL Queen Square Institute of Neurology, London, WC1N 3BG, United Kingdom
6. Queen Square Movement Disorders Centre, UCL Queen Square Institute of Neurology, London, WC1N 3BG, United Kingdom
7. Department of Neurodegenerative Disease, UCL Queen Square Institute of Neurology, London, WC1N 3BG, United Kingdom
8. Division of Neuropathology, National Hospital for Neurology and Neurosurgery, University College London NHS Foundation Trust, London, NW1 2PB, United Kingdom
9. UK Dementia Research Institute at The University of Cambridge, Cambridge, CB2 0AH, United Kingdom
10. Centre for Preventive Neurology, Wolfson Institute of Population Health, Queen Mary University London, London, E1 4NS, United Kingdom

Correspondence to: Zane Jaunmuktane

Department of Clinical and Movement Neurosciences and Queen Square Brain Bank for Neurological Disorders, UCL Queen Square Institute of Neurology, University College London, London, WC1N 3BG, UK

E- mail: z.jaunmuktane@ucl.ac.uk

Correspondence may also be addressed to: Hemanth R. Nelvagal

E-mail: h.nelvagal@ucl.ac.uk

Supplementary methods

Immunohistochemical staining

215 of 2,057 Aβ sections (10.5%), 150 of 2,116 pTau sections (7.1%), and 304 of 3,141 α-synuclein sections (9.7%) required re-staining. Immunostaining was performed over multiple batches. 7 µm sections were cut from formalin-fixed, paraffin-embedded tissue blocks from representative brain regions and mounted on glass slides (see Supplementary Table 1 for a complete list of regions). Immunohistochemical staining for α-synuclein (MA1-90342; Thermo Scientific; 1:1,500), amyloid-β (M0872; Dako; 1:100), phosphorylated tau (MN1020; AT8; Thermo Scientific; 1:1,200) and non-phosphorylated TDP-43 (2E2-D3, H00023435-M01, Abnova, 1:500) was performed using automated staining platforms from Menarini Diagnostics or Ventana (Roche). Biotinylated secondary antibodies were used with horseradish peroxidase-conjugated streptavidin complex and diaminobenzidine as the chromogen, with haematoxylin counterstaining. Appropriate positive and negative controls were included in each staining run. Slides were also stained for haematoxylin & eosin. Pathological diagnoses were confirmed by neuropathologists experienced in neurodegenerative diseases using relevant consensus diagnostic and staging criteria.[1-7]

Quantitative digital neuropathology

Regions of interest (ROIs) were defined based on standard neuroanatomical landmarks. The full list of ROIs and their anatomical boundaries is provided in Supplementary Table 1. ROIs were manually delineated on digital whole-slide images using NZConnect (web-based) or NDP.view2 (offline) following the same anatomical landmark criteria across all cases. For each ROI, annotators outlined the relevant grey-matter region while excluding large vessels, artefacts, and tissue folds. In addition, a small circular annotation ("!BACKGROUND", diameter 10.5 µm) was placed in the area of highest visually apparent background DAB signal for each brain region to allow local background estimation and subsequent background-corrected thresholding.

The following image analysis methods were performed in QuPath v0.5.1 after importing annotations.[8]

a. Colour deconvolution and background correction:

To address batch effects in stain intensity, stain vectors for colour deconvolution were calculated from a composite image consisting of 1000×1000 pixels squares derived from a subset of 270 whole slide images (WSI), randomly selected from across the entire cohort and stained for α-synuclein, amyloid-β (Aβ) and phosphorylated tau (pTau), with 1% of extreme pixels ignored. This is to account for variability in haematoxylin and DAB staining intensities across the years that the slides were stained and to establish stain vectors that are specific for the colour profile of the slide scanners used in this study. After applying the stain vectors onto each WSI, to further address batch variations in staining intensity, the mean deconvoluted DAB intensity within each "!BACKGROUND" (BG) annotation was calculated (DAB: Mean (BG)) and saved as a measurement in their corresponding brain region annotation. The resulting DAB-channel specific images were then analysed for Aβ and pTau using automatic thresholding and for Lewy bodies using the deep learning-based segmentation algorithm *StarDist,*[9] respectively.

b. Lewy body classifier:

α-synuclein images were pre-processed similarly by importing WSI and ROI annotations and then applying stain vectors for colour deconvolution. To detect Lewy bodies, the deep learning-based segmentation algorithm *StarDist*[9] was used on the deconvoluted DAB channel. The resulting detections were dilated by 2 µm to create a pseudo-cell where the "nucleus" is the original detection and the "cytoplasm" is the area surrounding the "nucleus" after 2 µm dilation.

This is used to measure the background DAB intensity surrounding each detection object. For each pseudo-cell, the following shape and intensity measurements were measured: intensity, shape/morphology and Haralick as described in Supplementary Table 6.

These measurements were used to train a random trees object classifier from a set of 40 training images to classify Lewy bodies from non-specific detections. The resulting Lewy body object classifier was then applied to the entire cohort of WSIs. The Lewy body detections were then divided by the area to give measurements of Lewy bodies per mm^2 for each ROI (abbreviated as LB/mm^2 in Figures and Tables). Representative examples of positive detections are shown in Supplementary Fig. 7.

c. Aβ and pTau automatic thresholding:

For each brain region, a threshold for positive DAB staining was calculated using automatic thresholding methods Triangle[10] or Otsu[11] via an adaptation of FIJI/ImageJ's implementation (https://github.com/fiji/Auto_Threshold). Using the calculated background DAB intensity and the automatic threshold values for positive DAB staining, the following threshold values were derived:

$$Background\ corrected\ fixed\ threshold\ value = DAB{:}\ Mean\ (BG) + 0.25$$

$$Background\ corrected\ Triangle\ threshold\ value\ (Amyloid\ \beta) = DAB{:}\ Mean\ (BG) + Triangle\ threshold\ value$$

$$Background\ corrected\ Otsu\ threshold\ value\ (pTau) = DAB{:}\ Mean\ (BG) + Otsu\ threshold\ value$$

The derived background-corrected threshold values were then used to measure the area of positive DAB staining, Triangle[10] for Aβ and Otsu[11] for pTau, respectively, within each brain region annotation. Finally, the percentage of positive DAB staining within each brain region was calculated by:

$$\%\ positive\ DAB\ staining\ area = \frac{Area\ of\ positive\ DAB\ staining}{Area\ of\ brain\ region\ annotation} \times 100$$

These values were then plotted as either % area of Aβ (abbreviated as %Aβ in Figures and Tables) or % area of phosphorylated tau (abbreviated as %pTau in Figures and Tables), respectively. Representative examples of positive detections are shown seen in Supplementary Figs 8 and 9 for Aβ and pTau respectively.

To assess whether residual background DAB variability influenced quantitative pathology measures, we evaluated the association between background-corrected mean DAB intensity and corresponding pathology burden (LB/mm², %Aβ, %pTau) using correlation analyses and background-stratified comparisons (Supplementary Fig. 10).

Multivariate associations with dementia risk

Multivariable logistic regression models were fitted separately for APOE ε3 and ε4 carriers to assess independent associations between quantitative misfolded-protein burden (α-synuclein, Aβ, pTau), ischaemic pathology, demographic factors, and dementia status. Continuous predictors were entered as linear terms, and binary covariates were coded as described in the main Methods. Odds ratios (ORs) were obtained by exponentiating the regression coefficients, and 95% confidence intervals were derived from the standard errors of the model coefficients. Statistical significance was evaluated using two-sided Wald tests.

a. Model evaluation

Model discrimination, calibration, and classification accuracy were evaluated using AUC (pROC), Youden-optimised threshold (with sensitivity, specificity, and accuracy), Brier score, Hosmer–Lemeshow test (10 groups) and F1 score. For APOE ε3, model performance was: AUC 0.775, optimal threshold 0.535, sensitivity 0.786, specificity 0.696, accuracy 0.746, F1 0.775, Brier 0.190, and Hosmer–Lemeshow $P = 0.810$. For APOE ε4, performance was: AUC 0.917, threshold 0.442, sensitivity 0.933, specificity 0.750, accuracy 0.870, F1 0.903, Brier 0.113, and Hosmer–Lemeshow $P = 0.691$. All diagnostics are provided in Supplementary statistics.

b. Cumulative risk heatmap

To evaluate dementia risk across all combinations of key pathology and demographic predictors, we constructed a cumulative risk matrix using an exhaustive subgrouping approach based on seven binary variables: α-synuclein burden (high vs low, 75th percentile cutoff), Aβ burden (high vs low, 75th percentile cutoff), pTau burden (high vs low, 85th percentile cutoff), ischaemic pathology (present vs absent), biological sex (male vs female), disease duration (<15 vs ≥15 years), and age at death (<77 vs ≥77 years). These groupings were generated separately for APOE ε3 and ε4 carriers.

All 128 possible combinations of the seven binary variables were constructed using the expand.grid() function in R.[12] The resulting predicted probabilities were visualized in a heatmap matrix to illustrate how dementia risk varies across strata of pathology burden and demographic, and clinical parameters.

c. Rule-based surrogate decision tree

To enable interpretable subgroup-level stratification, a rule-based surrogate decision tree was constructed using seven binary predictors. APOE ε3 and ε4 groups were analysed separately, with cutoffs for α-synuclein and Aβ set at the 75th percentile and for pTau at the 85th percentile, calculated within each genotype group. For each of the 128 possible combinations of binary predictors, dementia probability was estimated using predictions from the logistic regression model. Variables were arranged in the tree according to decreasing magnitude of genotype-specific logistic regression model coefficients (odds ratios) for dementia risk, with the strongest predictors placed nearest the root. We evaluated model performance using ROC analysis with the pROC package in R,[13] reporting the area under the ROC curve (AUC) and the optimal probability threshold determined by Youden's J statistic,[14] together with the corresponding sensitivity and specificity values. All evaluations were performed separately within each genotype subgroup using stratified 10-fold cross-validation.

Regional quantitative pathology-driven disease progression modelling

SuStaIn simultaneously infers subtype-specific progression sequences and probabilistic subtype/stage assignments from cross-sectional data using a generative modelling framework.[15] Model uncertainty was assessed with 5,000,000 Markov Chain Monte Carlo (MCMC) iterations, and the expectation-maximization process optimised the subtype sequences from 30 random starting points to find the maximum likelihood solution. Model selection was guided by 10-fold cross-validation using the cross-validation information criterion (CVIC) at 5,000,000 MCMC iterations, with lower values indicating better fit, tested from 1-7 subtypes. We also examined test-set log-likelihoods to evaluate generalizability, and MCMC trace plots and histograms to confirm stability across subtypes. The optimal Lewy pathology model and combined Lewy, Aβ, and pTau pathology model supported 4 subtypes as, despite decreasing CVIC values with increasing complexity, there was little increase in test-set log-likelihoods (Supplementary Figs 3 and 5, Supplementary Data).[16] An equivalent SuStaIn

modelling procedure was applied to semiquantitative regional α-synuclein scores using the ordinal SuStaIn framework, which is specifically designed for ordered categorical pathology scores. Semiquantitative scores were modelled as monotonic severity transitions across regions, and the number of subtypes was fixed to four based on the optimal quantitative model to ensure direct comparability of inferred progression sequences. This analysis was intended to assess concordance of subtype-level progression patterns rather than exact correspondence of individual subject assignments (Supplementary Fig. 11). Each SuStaIn subtype in Figs 6, 7 and Supplementary Fig. 11 are visualized using a positional variance diagram, which illustrates the most probable sequence of regional involvement across SuStaIn stages, along with the associated uncertainty. Regional pathology values were standardised against the synthetic control distribution, such that the control mean defines the baseline (0) and each unit reflects one control standard deviation (SD). In the SuStaIn positional variance diagrams, three abnormality thresholds relative to controls are shown: mild (≥1 SD, red), moderate (≥2 SD, magenta), and severe (≥3 SD, blue). Colour intensity indicates the certainty with which the model assigns a biomarker to a given abnormality threshold at each disease stage: deeper hues denote higher certainty, paler hues indicate greater positional variance (uncertainty), and white reflect minimal or no probability. Subtype assignments were generated using the standard SuStaIn implementation (available at https://github.com/neurolibre/pysustain), with all analyses run on identical regional inputs to ensure comparability across models. Stability of subtype solutions was confirmed by examining MCMC convergence and by verifying reproducible subtype frequencies across cross-validation folds.

Statistical analysis

For pathology burden classification and diagnostic performance metrics, quantitative measures of each misfolded proteinopathy (α-synuclein, Aβ, and pTau) were compared between binarised subgroups using the Kruskal–Wallis test followed by Dunn's pairwise post-hoc comparisons with Benjamini–Hochberg (BH)[17] *P*-value correction. Spearman's rank correlation was used to evaluate the association between semi-quantitative pathology scores and their corresponding quantitative burdens (Spearman ρ: α-synuclein = 0.72, Aβ = 0.80, pTau = 0.76; Spearman $P < 0.0001$ for all three pathologies), as visualized in scatter plots in Fig. 1. Kruskal-Wallis test with Dunn-BH correction was employed to compare: (i) traditional pathology staging schemes vs. quantitative misfolded protein pathological burdens (Fig. 1) and

(ii) regional pathology across quantitative misfolded protein pathologies (Figs 2A, 2B, 3, Supplementary Figs 1, 4 and 6, Supplementary Data).

Two-sided Kolmogorov-Smirnov tests were used to compare the distribution of quantitative pathology burden between APOE ε3 and ε4 carriers (Fig. 2C, Supplementary Data). Chi-square or Fisher's exact tests (when expected counts were <5) were used to compare proportions of dementia cases across semi-quantitatively determined pathology stages (Braak stages for Lewy pathology, Thal phases for Aβ and Braak & Braak stages for pTau) and quantitative pathological groupings (Fig. 2D, Supplementary Data) and all categorical clinical and demographic variables (Table 1, Supplementary Tables 3, 4 and 5, Sankey diagrams for Figs 6 and 7, and Supplementary Data). Mann-Whitney U tests with BH correction were used to compare regional quantitative Lewy, Aβ and pTau pathology between APOE ε3 and ε4 genotypes (Fig. 3, Supplementary Data) and between PD cases with and without dementia (Supplementary Fig. 2). A binary univariate logistic regression model was fitted for the presence and absence of ischaemic pathology, separately for cases with low and high misfolded protein pathology burden respectively (Fig. 4B) and a multivariate generalized linear model (glm) was fitted separately for APOE ε3 and ε4 carriers for the following variables: α-synuclein burden, Aβ burden, pTau burden, ischaemic pathology (present vs absent), biological sex (male vs female), disease duration (<15 vs ≥15 years), and age at death (<77 vs ≥77 years) and significance was assessed using Wald tests on the model coefficient for each variable (Fig. 5A and Supplementary Data). For both regression analyses, the outcome variable was the presence or absence of dementia.

To evaluate if residual staining/background variability could bias quantitative pathology estimates, we tested the association between background-corrected mean DAB intensity (computed per ROI from background annotations after colour deconvolution) and the corresponding quantitative pathology burden (LB/mm^2, %Aβ area, %pTau area respectively) using Spearman's rank correlation and linear regression (Supplementary Fig. 10). We additionally compared background DAB distributions across pathology-burden strata (high vs low; median split) and across background-intensity quartiles to assess potential systematic shifts (Supplementary Fig. 10).

Supplementary figures and legends:

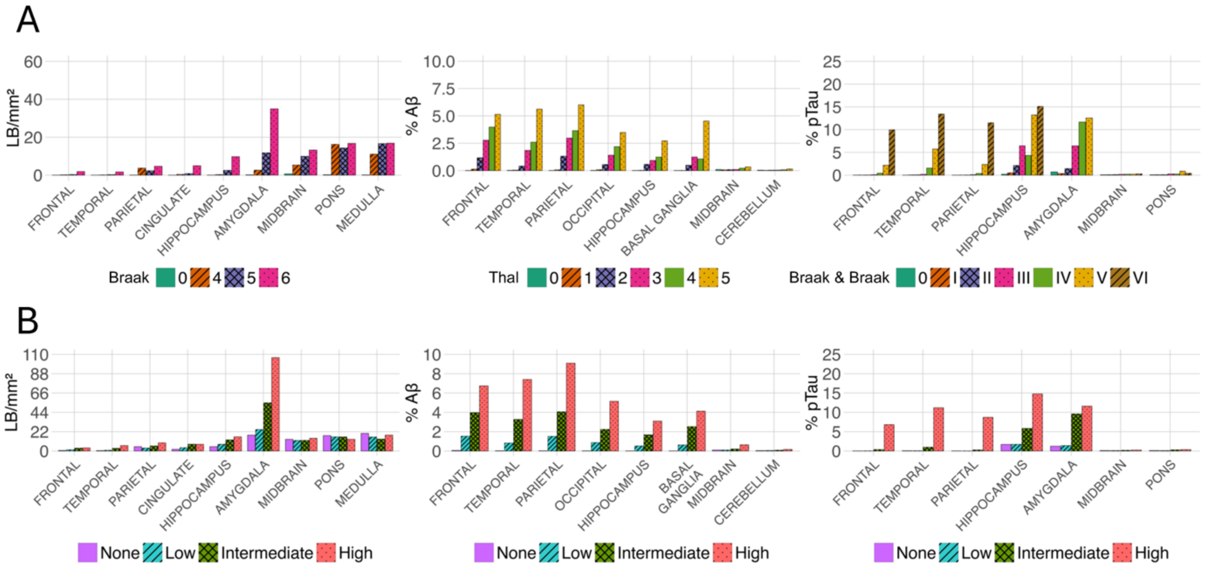

Supplementary Figure 1: Histogram plots of regional quantitative pathology burden stratified by traditional staging.

(A) Quantitative pathology burden across brain regions stratified by stage or phase of the corresponding misfolded proteinopathy.

Histogram plots show the quantitative pathology burden (y-axis) of Lewy bodies (LB/mm^2), % area of Aβ (% Aβ), and % area of phosphorylated tau (%pTau) across anatomical brain regions (x-axis), stratified by stage or phase of the respective proteinopathy. Lewy body burden plotted by Braak stage (0, 4, 5, 6). Aβ burden stratified by Thal phase (0–5). pTau burden stratified by Braak & Braak stage (0–VI). Detailed statistical outputs are available in supplementary Excel statistics data for Fig. 2A. Kruskal-Wallis tests with Dunn's pairwise post-hoc comparisons and Benjamini-Hochberg (BH) adjustment was performed per region for each proteinopathy.

(B) Quantitative pathology burden across brain regions stratified by level of Alzheimer's disease neuropathologic change.

Histogram plots show the quantitative pathology burden (y-axis) of Lewy pathology (LB/mm^2), % area of Aβ (% Aβ), and % area of phosphorylated tau (%pTau) across anatomical brain regions (x-axis), stratified by Alzheimer's disease neuropathological change (ADNC) level as defined by NIA-AA criteria (none, low, intermediate, high). Detailed statistical outputs are available in supplementary Excel statistics data for Fig. 2B. Kruskal-Wallis tests with Dunn's pairwise post-hoc comparisons and Benjamini-Hochberg (BH) adjustment was

performed per region for each proteinopathy. Brain regions included are available in supplementary Excel statistics data for Fig 2B.

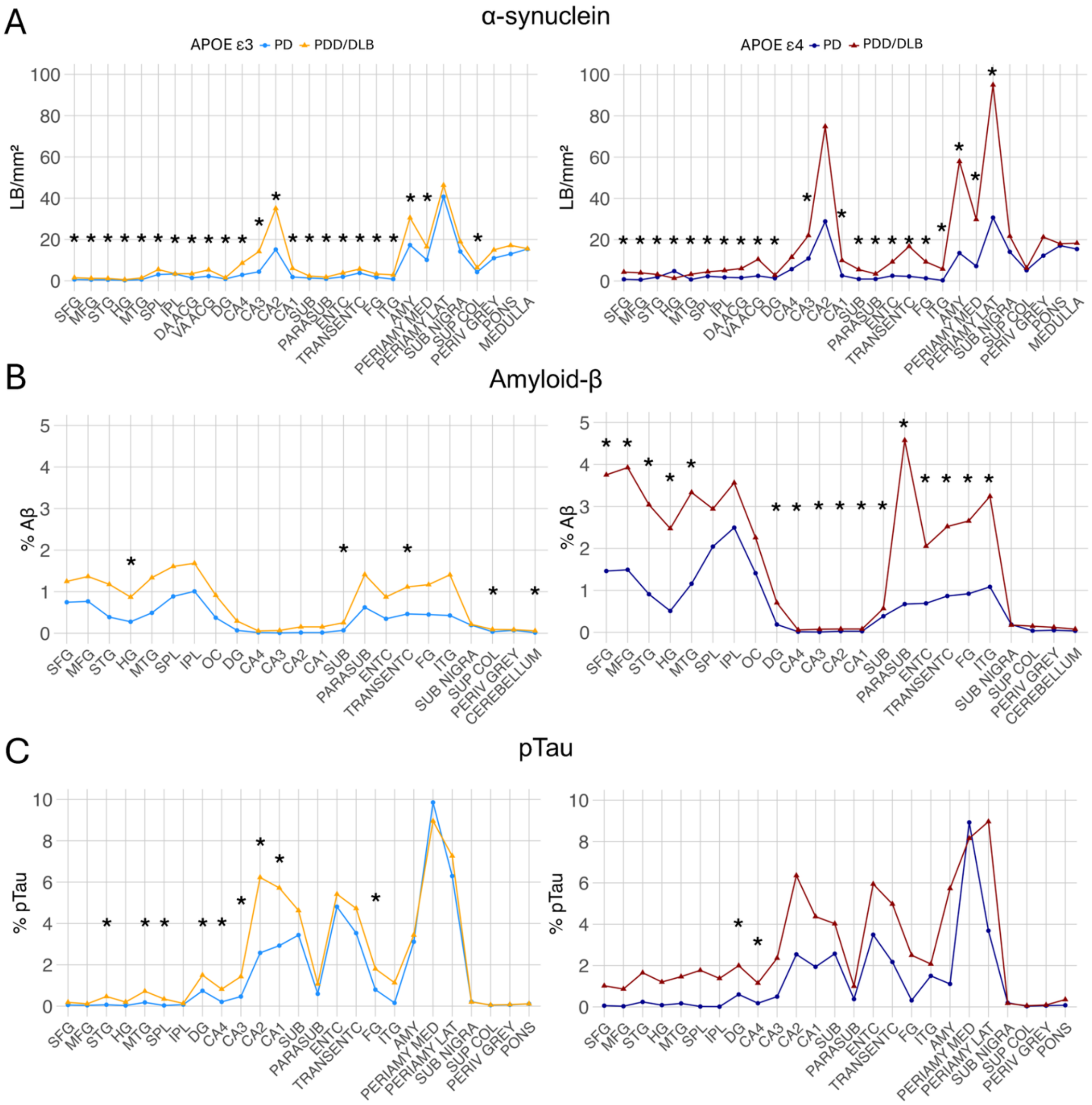

Supplementary Figure 2: Influence of dementia status on regional α-synuclein, Aβ and pTau pathology in Lewy body disease stratified by APOE genotype.

(A) Lewy body (LB/mm^2), **(B)** % area of Aβ (%Aβ) and **(C)** % area of phosphorylated tau (%pTau) quantitative pathology burden across multiple brain regions in APOE ε3 and ε4 carriers, stratified by dementia status (light blue APOE ε3 PD, yellow APOE ε3 PDD/DLB, dark blue APOE ε4 PD, brown APOE ε4 PDD/DLB).

Lines indicate group mean pathology burden across regions; * indicates statistically significant differences between dementia (PD vs PDD/DLB) groups in each region ($P < 0.05$; statistical

test Mann-Whitney test with BH correction per region for each proteinopathy). Brain region abbreviations are detailed in Supplementary Table 1 and detailed statistical outputs are available in supplementary data (Excel statistics) for Supplementary Figure 2.

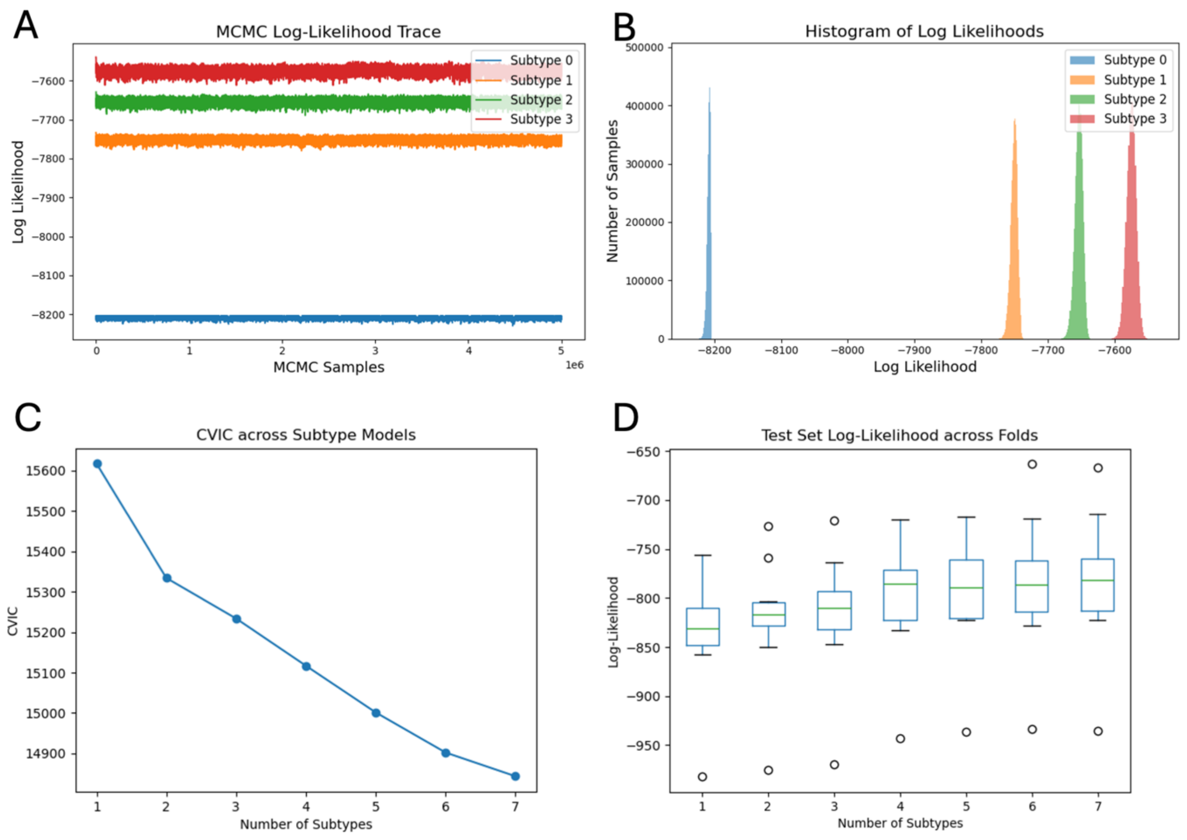

Supplementary Figure 3: SuStaIn modelling of regional Lewy pathology burden.

(A) Markov Chain Monte Carlo (MCMC) log-likelihood traces for four subtypes, showing stable convergence across 5 million iterations based on regional Lewy pathology. **(B)** Histograms of log-likelihood values for the four subtypes, illustrating the distribution across MCMC iterations. **(C)** Ten-fold cross-validation information criterion (CVIC) values for models with one to seven subtypes, with lower CVIC values indicating better fit. **(D)** Boxplots of test-set log-likelihood across ten cross-validation folds for one to seven subtype models, demonstrating model generalisability. Detailed outputs are available in supplementary data (Excel statistics) for Supplementary Figure 2.

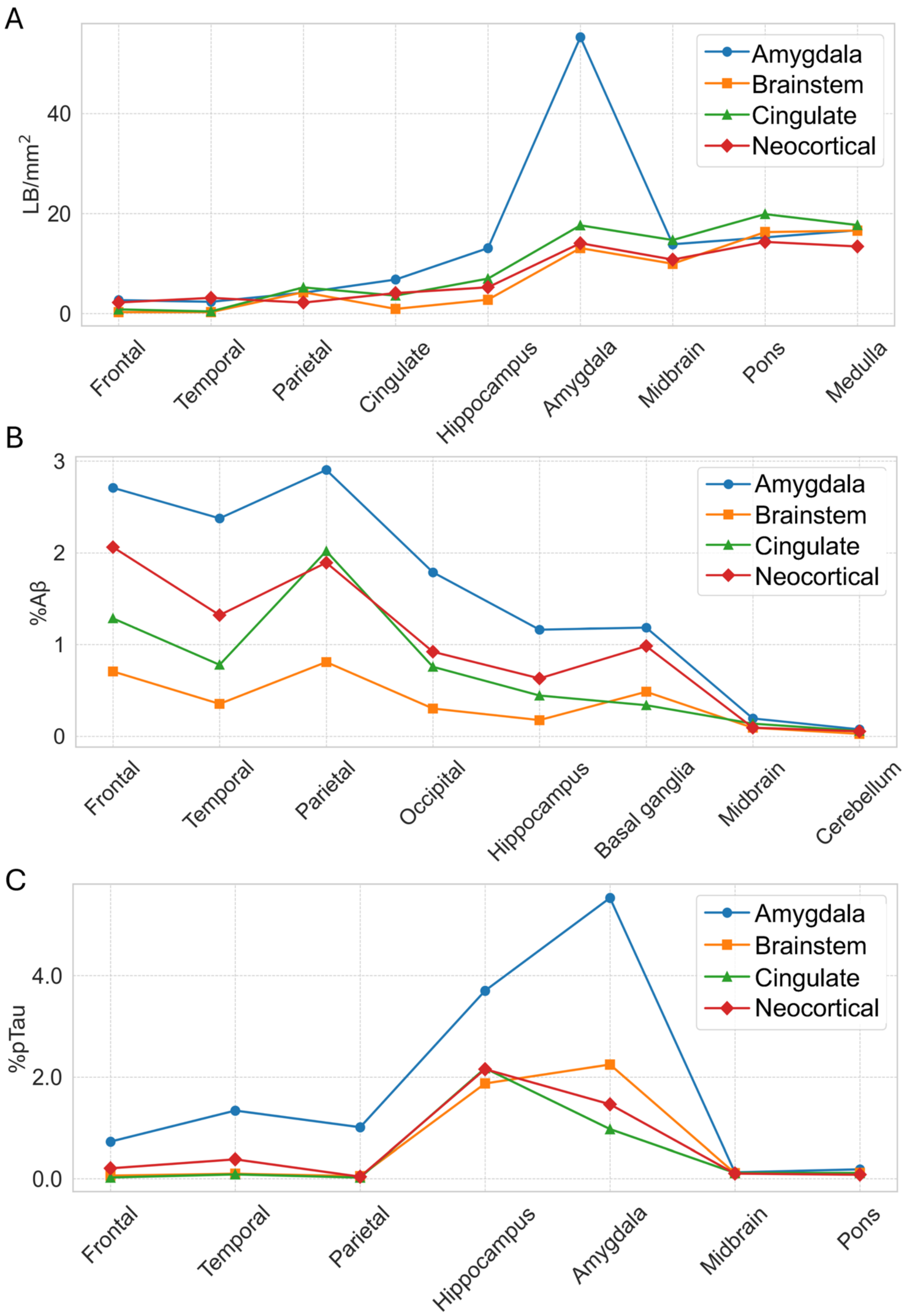

Supplementary Figure 4: Mean regional Lewy, Aβ, and pTau pathology across SuStaIn subtypes of Lewy pathology progression.

Each line shows mean quantitative **(A)** Lewy body (LB/mm^2), **(B)** % area of Aβ (%Aβ) and **(C)** % area of phosphorylated tau (%pTau) pathology burden across multiple brain regions, stratified by SuStaIn-inferred subtypes of Lewy pathology progression based on quantitative Lewy body burden. Each line represents the mean pathology burden for a subtype across regions. Brain regions include average quantitative scores from frontal (cortices of the superior frontal and middle frontal gyri), temporal (cortices of the superior temporal, Heschl's and middle temporal gyri), parietal (cortices of the superior parietal and inferior parietal lobules), occipital (occipital cortex), medial temporal (dentate gyrus, CA4, CA3, CA2, CA1, subiculum, parasubiculum, entorhinal cortex, transentorhinal cortex, cortex of fusiform gyrus), amygdala, basal ganglia (caudate nucleus and putamen) midbrain (substantia nigra, superior colliculus, periventricular grey matter), pons (pontine tegmentum), medulla (medullary tegmentum) and cerebellum. Detailed statistical outputs for Kruskal-Wallis test with Dunn's pairwise post-hoc comparisons and Benjamini-Hochberg (BH) *P*-value adjustment in supplementary data (Excel statistics) for Supplementary Figure 3.

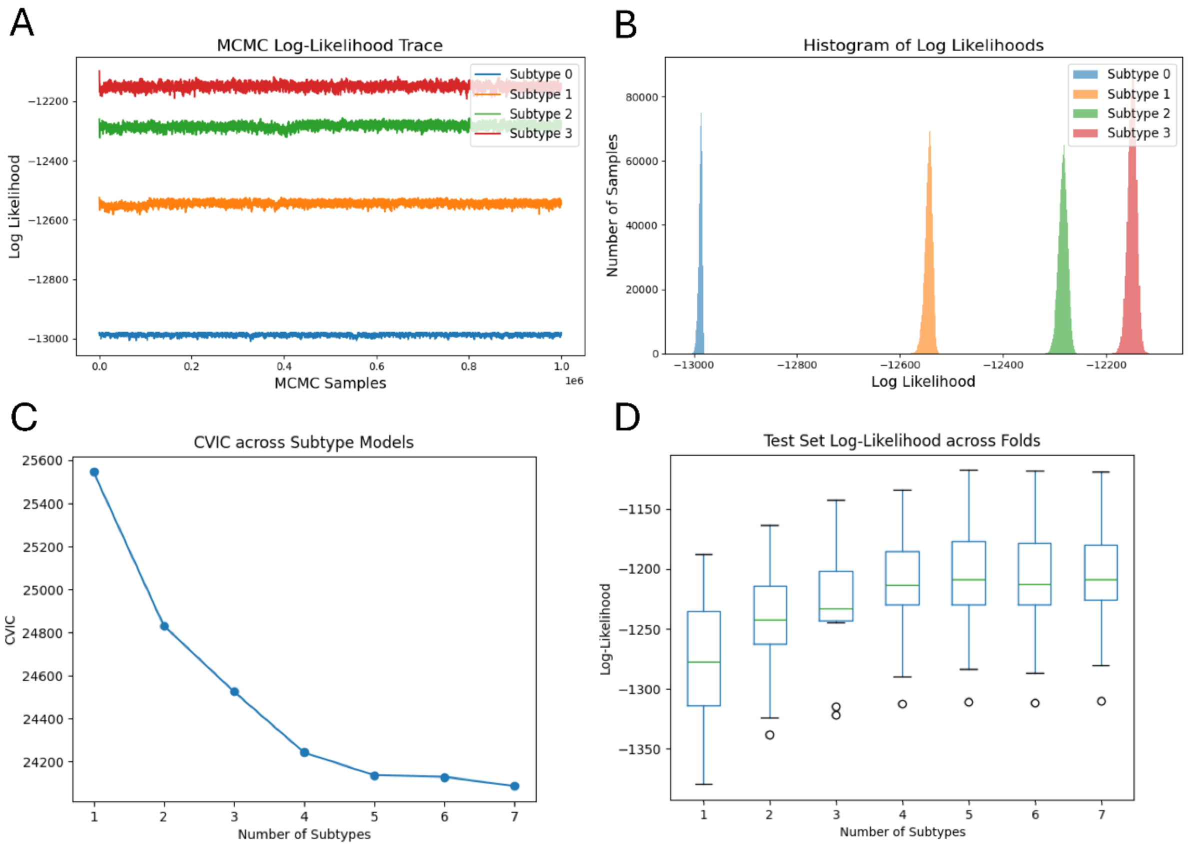

Supplementary Figure 5: SuStaIn modelling of combined Lewy, Aβ, and pTau pathology.

(A) Markov Chain Monte Carlo (MCMC) log-likelihood traces for four subtypes based on frontal cortical and medial temporal lobe quantitative Lewy, Aβ, and pTau pathology, showing

stable convergence across 5 million samples. Cortical regions include cortices of the superior and middle frontal gyri, cortices of the superior and middle temporal and Heschl's gyri, and cortices of the superior and inferior parietal lobules; medial temporal lobe regions include dentate gyrus, CA4, CA3, CA2, CA1, subiculum, parasubiculum, entorhinal cortex, transentorhinal cortex and cortex of the fusiform gyrus. **(B)** Histograms of log-likelihood values for the four subtypes, illustrating the distribution across MCMC iterations. **(C)** Ten-fold cross-validation information criterion (CVIC) values for models with one to seven subtypes, with lower CVIC values indicating better fit. **(D)** Boxplots of test-set log-likelihood across ten cross-validation folds for one to seven subtype models, demonstrating model generalisability. Detailed outputs are available in supplementary data (Excel statistics) for Supplementary Figure 4.

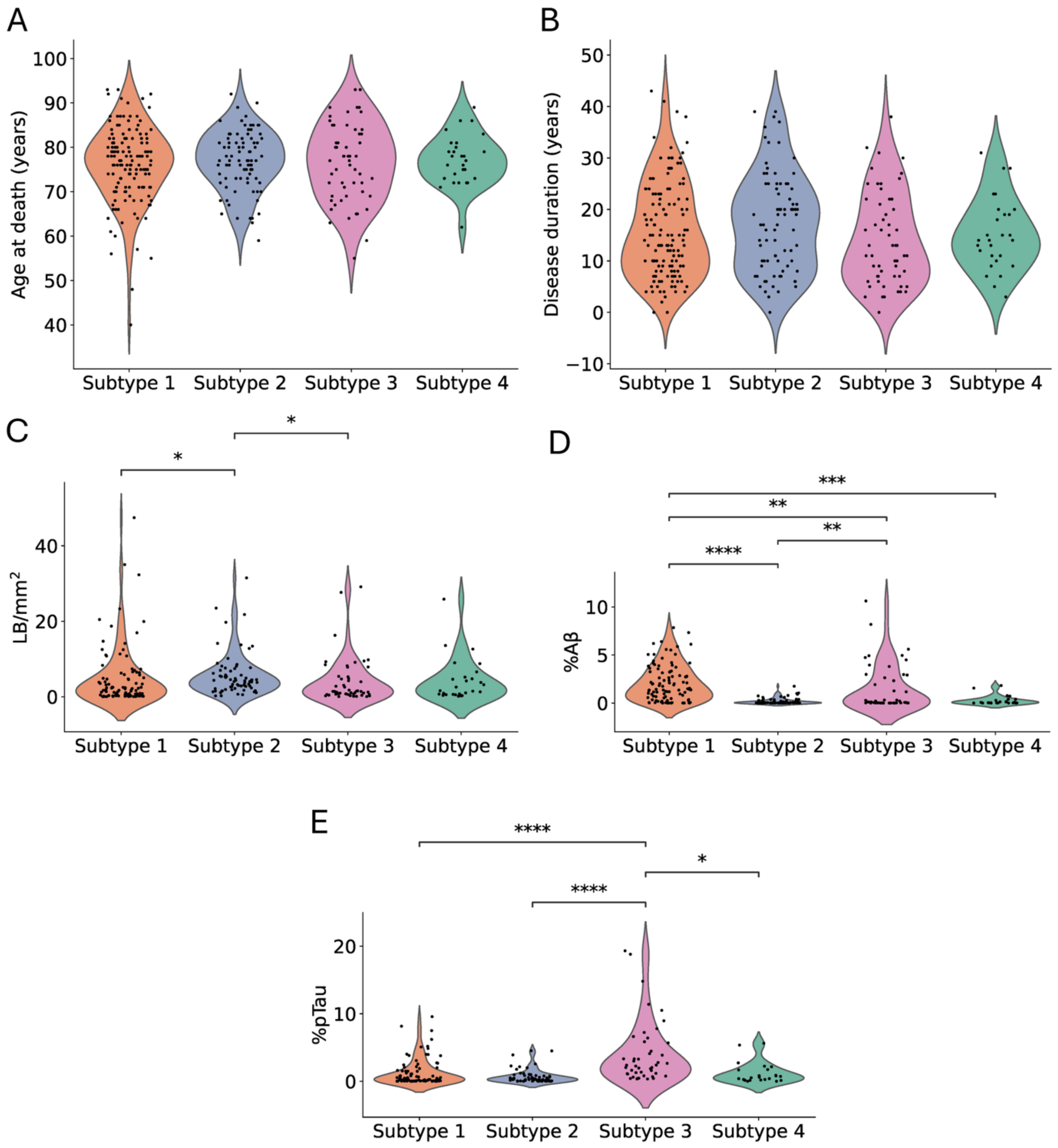

Supplementary Figure 6: Demographics and regional pathology distribution across SuStaIn subtypes of combined Lewy, Aβ, and pTau progression.

(A) Age at death (years) and **(B)** disease duration (years) for SuStaIn-inferred spatiotemporal trajectories. **(C)** Lewy body (LB/mm^2), **(D)** % area of Aβ (%Aβ) and **(E)** % area of phosphorylated tau (%pTau) quantitative pathology burden across frontal cortical and medial temporal lobe regions, stratified by SuStaIn-inferred spatiotemporal progression subtypes of combined Lewy body density (LB/mm²), %Aβ and %pTau pathology. Frontal regions include cortices of the superior and middle frontal gyri and medial temporal regions include dentate gyrus, CA4, CA3, CA2, CA1, subiculum, parasubiculum, entorhinal cortex, transentorhinal

cortex and cortex of the fusiform gyrus. Kruskal-Wallis test with Dunn's pairwise post-hoc comparisons and Benjamini-Hochberg (BH) *P*-value adjustment, (* $P \leq 0.05$, ** $P \leq 0.01$, *** $P \leq 0.001$, **** $P \leq 0.0001$). Detailed outputs are available in supplementary data (Excel statistics) for Supplementary Figure 5.

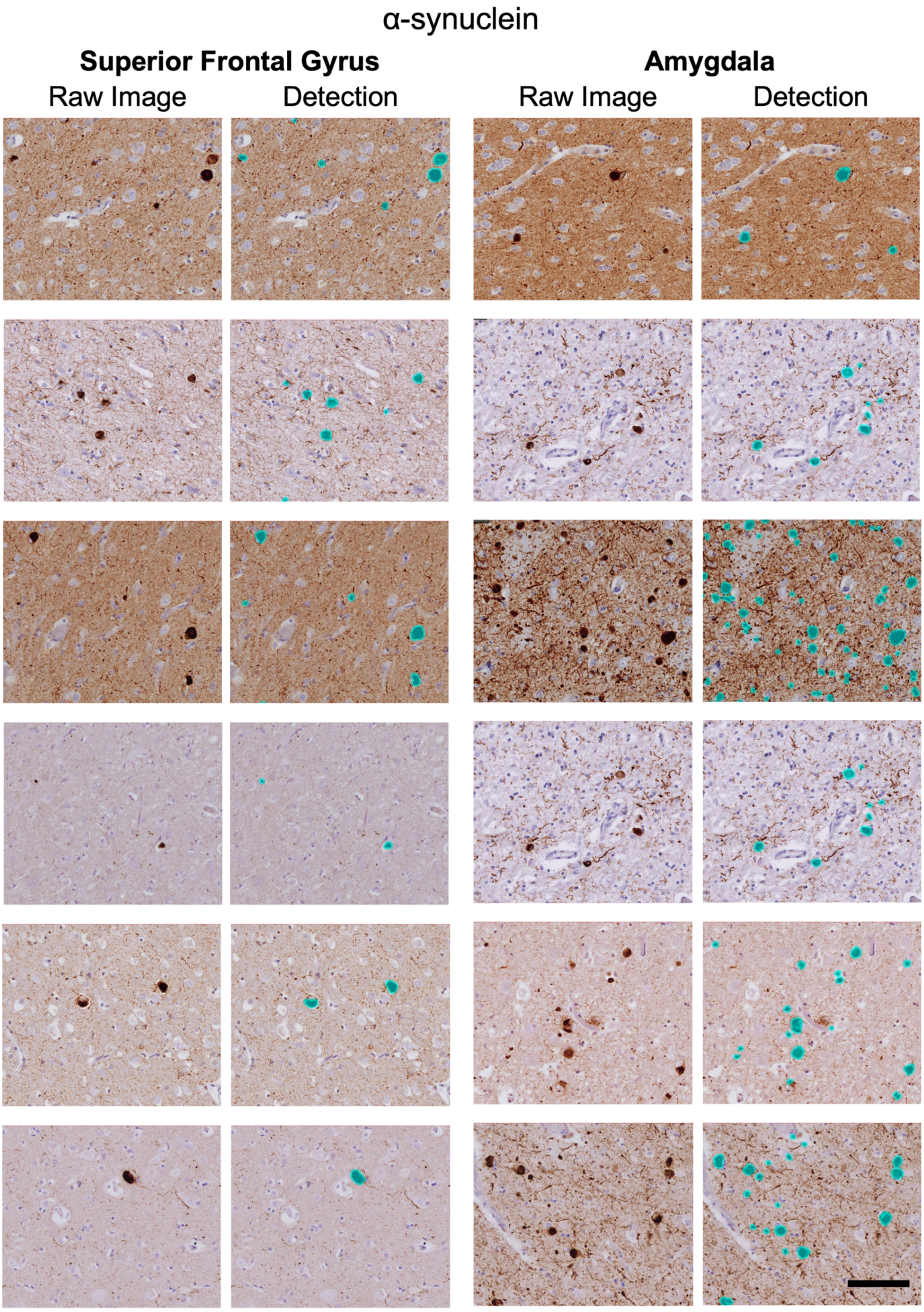
α-synuclein
Superior Frontal Gyrus
Amygdala
Raw Image
Detection
Raw Image
Detection

Supplementary Figure 7: Representative images of α-synuclein immunoreactive Lewy body detection.

Representative images from the cortex of superior frontal gyrus and amygdala showing raw 4D6 α-synuclein immunostaining (left column of each region) and corresponding automated Lewy body detections overlaid on the same fields (right column). Images are selected to illustrate the range of staining intensities, background variability, and Lewy body burden observed across the archival cohort, and demonstrate consistent performance of the detection algorithm across cortical and limbic regions despite heterogeneous synaptic background staining. Scale bar = 100 μm.
