# Supplementary material for: Quantitative pathology and APOE genotype reveal dementia risk and progression in Lewy body disease
Source: Brain. 2026 Mar 27;149(11):3905–22. doi: 10.1093/brain/awag114 (PMC13730591; doi:10.1093/brain/awag114)
Supplement: Supplementary file 1 [file awag114_supplementary_data.zip › brain-2025-02697-File011.pdf]

Quantitative pathology and APOE genotype reveal dementia risk and progression in Lewy body disease

Authors

Hemanth R Nelvagal[1,2,3], Nancy Chiraki[1,2,3], Toby Curless[3,4], Patrick W Cullinane[1,2,5,6], Alice Rockliffe[1,2], Samarth Pimparkar[1,2], Haruka Kawamura[2], Sophie Ollerenshaw[2], Isha Elahi[2], Sebastian Brandner[7,8], Lesley Wu[1], Raquel Real[1,3], Mina Ryten[3,9], John Hardy[3,7], Eduardo De Pablo Fernandez[1,2,5,10], Thomas T Warner[1,2,5,6], Huw R Morris[1,3,6], Yau Mun Lim[3,7], Zane Jaunmuktane[1,2,3,8]

Affiliations

1. Department of Clinical and Movement Neurosciences, UCL Queen Square Institute of Neurology, University College London, London, WC1N 3BG, United Kingdom
2. Queen Square Brain Bank for Neurological Disorders, UCL Queen Square Institute of Neurology, London, WC1N 1PJ, United Kingdom
3. Aligning Science Across Parkinson's (ASAP) Collaborative Research Network, Chevy Chase, MD 20815, USA
4. Department of Neuromuscular Diseases, UCL Queen Square Institute of Neurology, University College London, London, WC1N 3BG, United Kingdom
5. Reta Lila Weston Institute of Neurological Studies, UCL Queen Square Institute of Neurology, London, WC1N 3BG, United Kingdom
6. Queen Square Movement Disorders Centre, UCL Queen Square Institute of Neurology, London, WC1N 3BG, United Kingdom
7. Department of Neurodegenerative Disease, UCL Queen Square Institute of Neurology, London, WC1N 3BG, United Kingdom
8. Division of Neuropathology, National Hospital for Neurology and Neurosurgery, University College London NHS Foundation Trust, London, NW1 2PB, United Kingdom
9. UK Dementia Research Institute at The University of Cambridge, Cambridge, CB2 0AH, United Kingdom
10. Centre for Preventive Neurology, Wolfson Institute of Population Health, Queen Mary University London, London, E1 4NS, United Kingdom

Correspondence to: Zane Jaunmuktane

Department of Clinical and Movement Neurosciences and Queen Square Brain Bank for Neurological Disorders, UCL Queen Square Institute of Neurology, University College London, London, WC1N 3BG, UK

E- mail: z.jaunmuktane@ucl.ac.uk

Correspondence may also be addressed to: Hemanth R. Nelvagal

E-mail: h.nelvagal@ucl.ac.uk

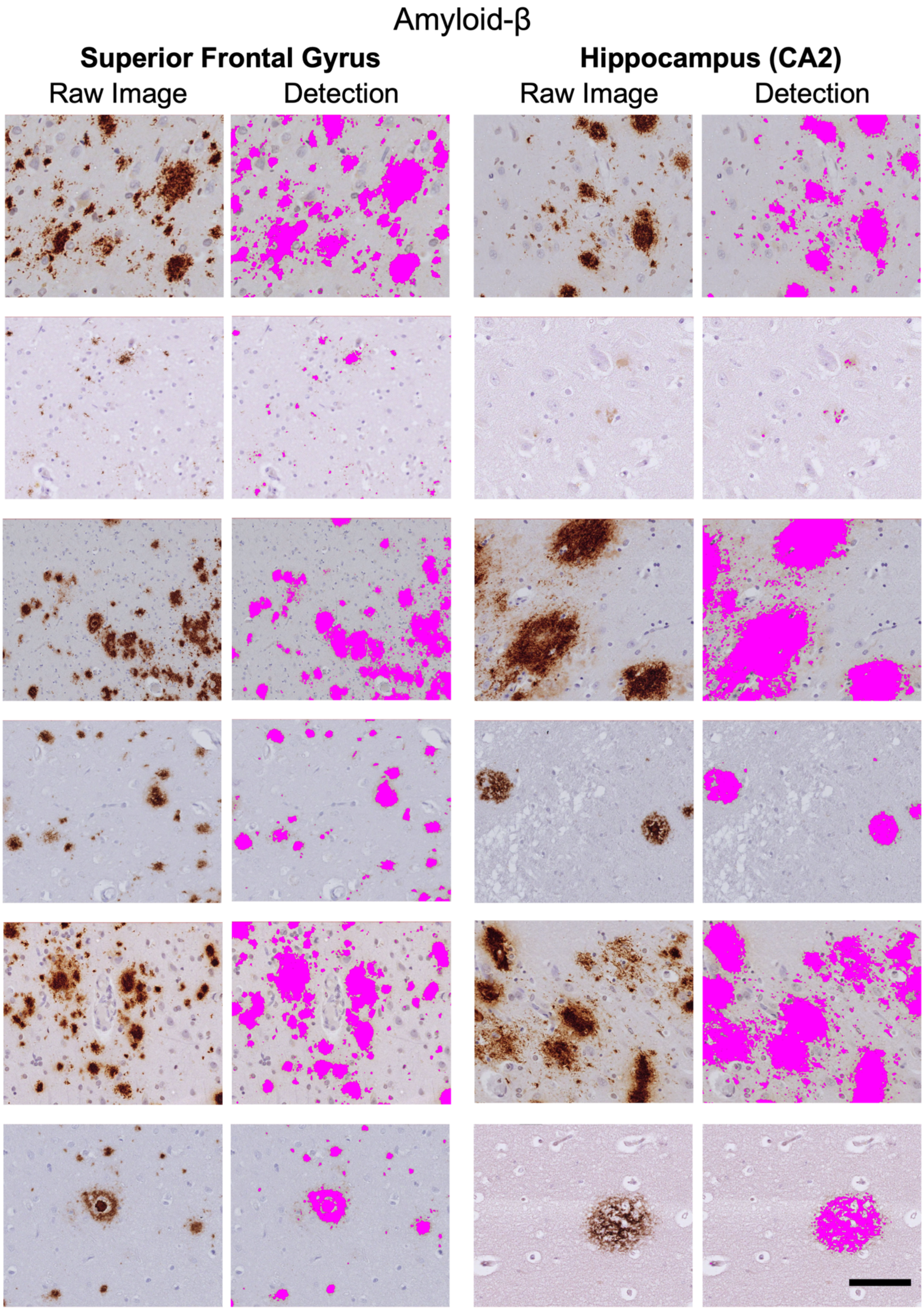

Supplementary Figure 8: Representative images of Aβ detection.

Representative images from the cortex of superior frontal gyrus and hippocampal CA2 region showing raw Aβ immunostaining (left column of each region) and automated plaque detections overlaid on the same fields (right column). Examples illustrate the algorithm's ability to identify Aβ pathology across a wide range of plaque morphologies, staining intensities, and background signal levels typical of archival material. Scale bar = 100 μm.

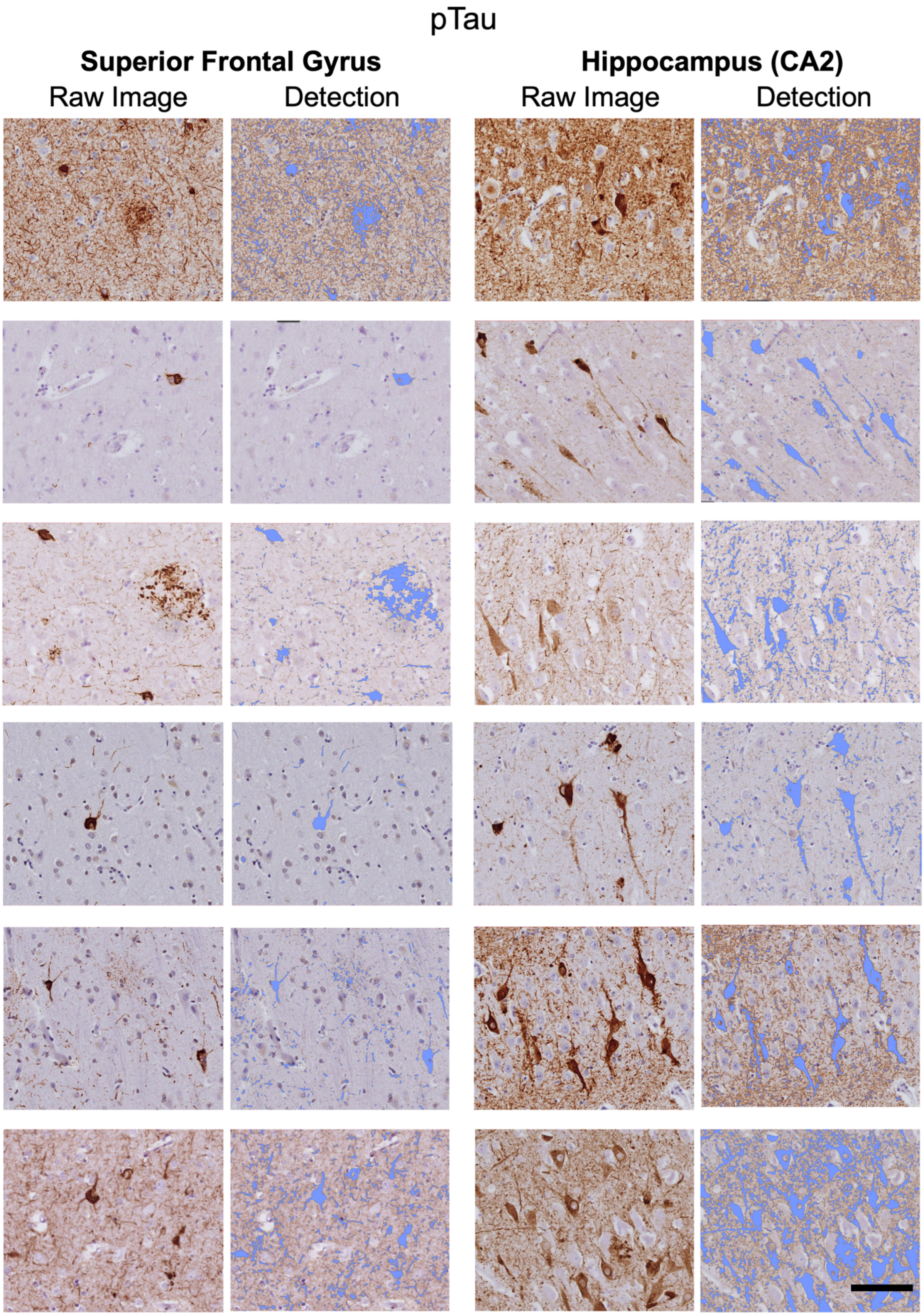

Supplementary Figure 9: Representative images of pTau detection.

Representative images from the cortex of superior frontal gyrus and hippocampal CA2 region showing raw pTau (AT8) immunostaining (left column of each region) and corresponding automated detections overlaid on the same fields (right column). Images highlight algorithm performance across cases with low to high pathological burden and varying background intensities, demonstrating robustness to staining heterogeneity. Scale bar = 100 µm.

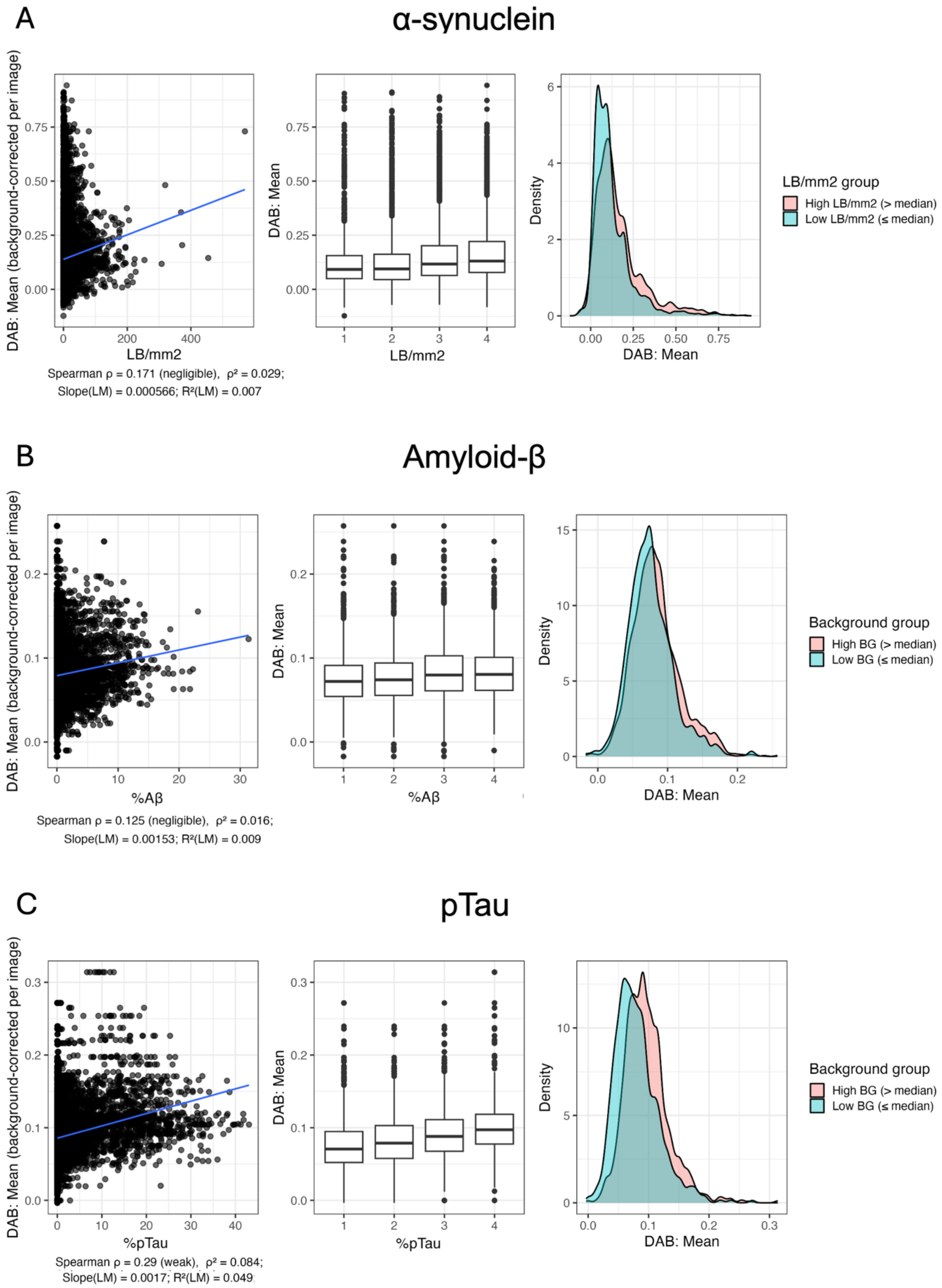

Supplementary Figure 10. Validation of background correction across immunohistochemical stains.

(A) α-synuclein, (B) Aβ, (C) pTau (pTau).

For each stain, three complementary analyses are shown to assess the relationship between background intensity and quantified DAB signal across the full dataset.

Left panels: Scatter plots showing the relationship between background metrics and mean DAB intensity per image. For α-synuclein, DAB signal is plotted against Lewy body density (LB/mm^2); for Aβ and pTau, DAB signal is plotted against background estimates derived from Triangle or Otsu thresholding. Blue lines indicate linear regression fits. Spearman correlation coefficients (ρ), effect sizes (ρ^2), regression slopes, R^2 values, and sample sizes (n) are indicated. In all cases, correlations are negligible to weak, explaining only a small fraction of variance in DAB intensity.

Middle panels: Boxplots showing mean DAB intensity stratified by quartiles of the corresponding background metric (quartile 1 = lowest background). While small shifts in median DAB values are observed across quartiles, extensive overlap between groups indicates that background contributes minimally to overall signal variation.

Right panels: Kernel density plots comparing DAB intensity distributions between high- and low-background groups (defined by median split). Distributions show substantial overlap, further demonstrating that background intensity does not drive DAB signal differences.

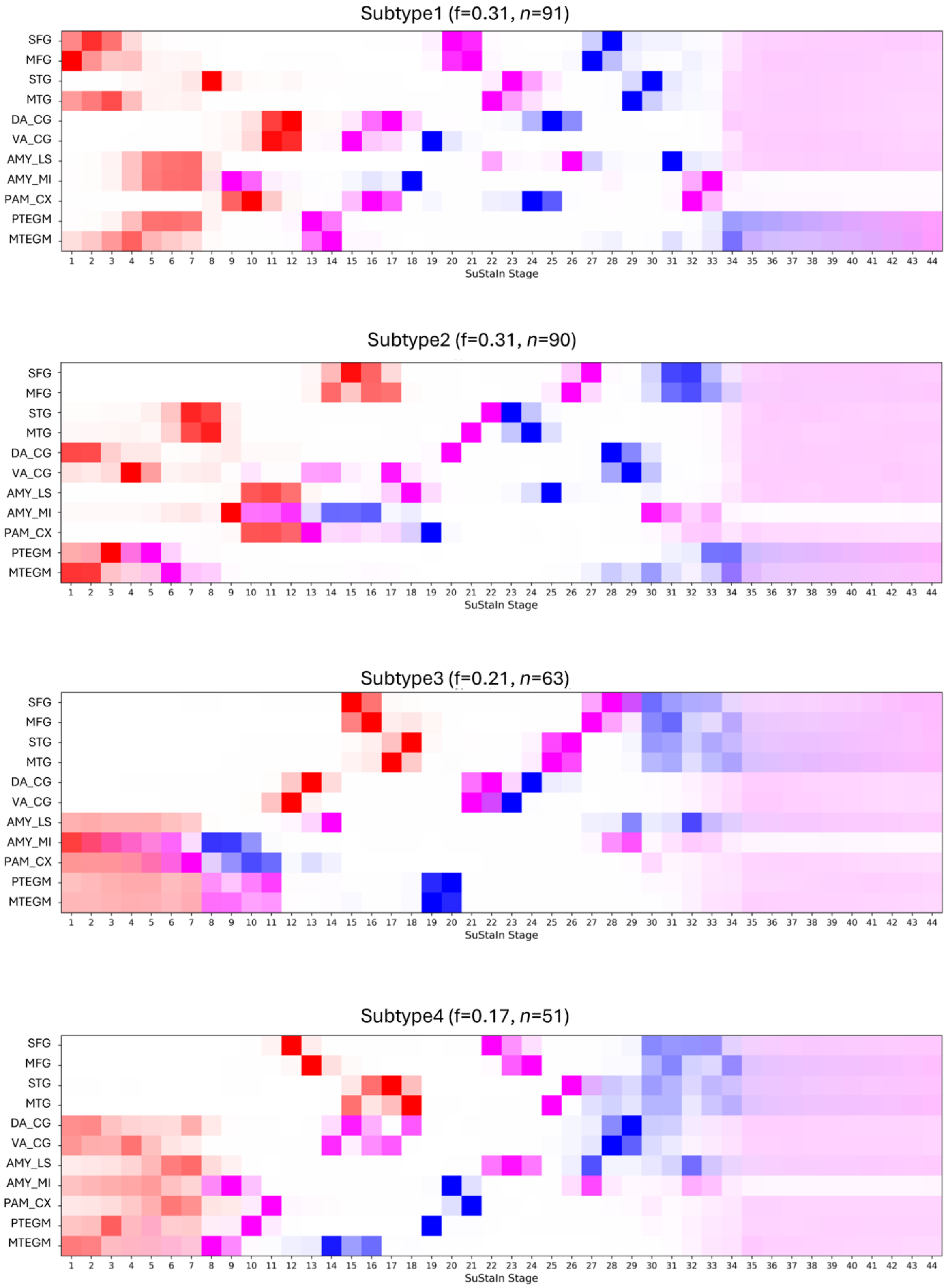

Supplementary Figure 11. Semiquantitative α-synuclein SuStaIn progression patterns.

Positional variance diagrams showing subtype-specific progression patterns derived from ordinal SuStaIn modelling of semiquantitative regional α-synuclein scores, with the number of subtypes fixed to four to enable direct comparison with the quantitative SuStaIn analysis. Rows

correspond to brain regions and columns to SuStaIn stages. Colours indicate increasing abnormality relative to the semiquantitative control distribution (mild, moderate, severe), with colour intensity reflecting model certainty at each stage. The semiquantitative SuStaIn analysis reproduces cingulate together with brainstem- and neocortical -predominant progression patterns consistent with the quantitative model, while a clearly brainstem-restricted subtype is not resolved, likely reflecting compressed dynamic range and relative overestimation of cortical pathology inherent to semiquantitative scoring. These results provide independent validation of the cortical-predominant trajectories identified using quantitative pathology.

Supplementary tables:

Supplementary Table 1: Abbreviations for regions of interest.

Region abbreviation	Anatomical region
**Frontal Cortex**	
SFG	Superior Frontal Gyrus (Grey Matter)
MFG	Middle Frontal Gyrus (Grey Matter)
**Temporal Cortex**	
STG	Superior Temporal Gyrus (Grey Matter)
MTG	Middle Temporal Gyrus (Grey Matter)
HG	Heschl's Gyrus (Grey Matter)
**Parietal Cortex**	
SPL	Superior Parietal Lobule (Grey Matter)
IPL	Inferior Parietal Lobule (Grey Matter)
**Occipital Cortex**	
OC	Occipital (Grey Matter)
**Cingulate Cortex**	
VA ACG	Ventro-Anterior Cingulate Cortex (Grey Matter)
DA ACG	Dorso-Anterior Cingulate Cortex (Grey Matter)
**Hippocampus**	
DG	Dentate Gyrus
CA4	CA4
CA3	CA3
CA2	CA2
CA1	CA1
SUB	Subiculum
PARASUB	Parasubiculum
ENTC	Entorhinal Cortex
TRANSENTC	Transentorhinal Cortex
FG	Fusiform Gyrus
ITG	Inferior Temporal Gyrus
**Amygdala**	
AMY	Amygdala
AMY_LS	Amygdala Lateral
AMY_MI	Amygdala Medial
PAM_CX	Periamygdaloid Cortex
**Midbrain**	
PERIV GREY	Periventricular Grey Matter
SUP COL	Superior Colliculus
SUB NIGRA	Substantia Nigra
**Pons**	
PONS/PTEGM	Pontine Tegmentum
**Medulla**	
MEDULLA/MTEGM	Medulla Tegmentum
**Cerebellum**	
CC	Cerebellar Cortex

List of abbreviations used to denote specific neuroanatomical regions of interest that are analysed for regional semi-quantitative and quantitative digital pathology.

Supplementary Table 2: Variables included in radar plots (Figure 1).

**Metric**	**Equation**	**Description**
Sensitivity	Sensitivity = TP / (TP + FN)	Proportion of dementia cases with high pathology correctly identified (true positive rate)
Specificity	Specificity = TN / (TN + FP)	Proportion of non-dementia cases with low pathology correctly identified (true negative rate)
Odds Ratio (OR)	OR = (TP × TN) / (FP × FN)	Measure of association between dementia status and pathology burden
Positive Predictive Value (PPV)	PPV = TP / (TP + FP)	Probability of dementia when pathology is high
Pathology Absence Ratio (PAR)	PAR = Dementia Low Path / Total Low Path	Proportion of dementia cases when pathology is low
Pathology Presence Ratio (PPR)	PPR = Dementia High Path / Total High Path	Proportion of dementia cases when pathology is high

TP = True Positives (dementia & high pathology), TN = True Negatives (no dementia & low pathology), FP = False Positives (no dementia & high pathology), FN = False Negatives (dementia & low pathology).

Supplementary Table 3. Clinical and demographic characteristics of Lewy body disease cohorts stratified by APOE status.

Cohort	n	Male, n (%)	Age at onset, years	Disease duration, years	Age at death, years	Dementia, n (%)	Orthostatic hypotension, n (%)	Ischaemic pathology, n (%)
APOE ε3	181	104/181 (57%)	61.2 ± 11.9	16.6 ± 9.5	77.6 ± 8.1	97/181 (54%)	58/84 (69%)	56/181 (31%)
APOE ε4	68	48/68 (71%)	60.5 ± 11.2	**13.9 ± 7.9***	**74.4 ± 7.7***	42/68 (62%)	28/35 (80%)	19/68 (28%)
**Cohort**	**Lewy body density (LB/mm^2)**	**%Aβ**	**%pTau**	**α-synuclein Braak stage[18]**	**Aβ Thal phase[19]**	**pTau Braak & Braak stage[1]**	**ADNC[4, 5]**	**TDP-43 LATE stage[6]**
APOE ε3	1.749 [0.011–47.453]	0.062 [0.001–7.846]	0.508 [0.005–14.81]	6 [0–6]	1 [0–5]	2 [0–6]	1 [0–3]	20/121 (17%)
APOE ε4	**4.533 [0.046–34.998]***	**1.351 [0.004–10.623]***	0.537 [0.007–18.803]	**6 [5–6]***	**3 [0–5]***	2 [0–6]	**1 [0–3]***	7/44 (16%)

Values are mean ± SD for age at onset, disease duration and age at death. Age at onset was calculated from reported symptom onset. Disease duration is symptom onset to death. Lewy body density, % area of Aβ (%Aβ), and % area of pTau (%pTau) are quantitative averages across defined cortical and medial temporal regions. Values for Lewy body density, %Aβ, and %pTau, α-synuclein Braak stage, Aβ Thal phase, pTau Braak & Braak stage, and Alzheimer's disease neuropathological change (ADNC) National Institute on Aging-Alzheimer's Association (NIA-AA) levels are median with [range]. ADNC: 0-3; Braak stage: 0-6; Aβ Thal: 0-5; pTau Braak & Braak stage: 0-VI (numbered 0-6). TDP-43 LATE staging represented as % cases, since all positive cases correspond to stage 2. *P*-values are Benjamini–Hochberg corrected values based on Fisher's exact tests, χ^2 proportion tests, or pairwise Mann–Whitney tests, as appropriate. Full statistics are provided in Supplementary Statistics Tables.

Bold text and superscripts indicate significant pairwise differences and correspond to the following significant contrast:

* $P<0.05$ for APOE ε3 vs APOE ε4

Supplementary Table 4. Clinical and demographic characteristics of Lewy body disease cohorts stratified by dementia and APOE status.

Cohort	n	Male, n (%)	Age at onset, years	Disease duration, years	Age at death, years	Dementia, n (%)	Orthostatic hypotension, n (%)	Ischaemic pathology, n (%)
PD APOE ε3	84	44/84 (52%)	61.3 ± 12.5	16.5 ± 9.5	77.3 ± 8.6	0/84 (0%)	24/37 (65%)	19/84 (23%)
PD APOE ε4	26	16/26 (62%)	60.4 ± 10.9	14.1 ± 5.3	74.5 ± 7.9	0/26 (0%)	10/13 (77%)	9/26 (35%)
PDD/DLB APOE ε3	97	60/97 (62%)	61.2 ± 11.3	16.7 ± 9.5	77.8 ± 7.6	**97/97 (100%)[a,b]**	34/47 (72%)	37/97 (38%)
PDD/DLB APOE ε4	42	32/42 (76%)	60.5 ± 11.5	13.8 ± 9.3	74.3 ± 7.6	**42/42 (100%)[a,b]**	18/22 (82%)	10/42 (24%)

Cohort	Lewy body density (LB/mm^2)	%Aβ	%pTau	α-synuclein Braak stage[18]	Aβ Thal phase[19]	pTau Braak & Braak stage[1]	ADNC[4, 5]	TDP-43 LATE stage[6]
PD APOE ε3	0.959 [0.011–12.627]	0.041 [0.002–4.954]	0.272 [0.005–7.23]	6 [0–6]	1 [0–4]	2 [0–4]	1 [0–2]	6/51 (12%)
PD APOE ε4	0.87 [0.046–21.779]	0.248 [0.004–4.143]	0.266 [0.007–2.686]	**6 [5–6][a]**	**3 [0–5][a]**	2 [0–4]	1 [0–2]	2/16 (13%)
PDD/DLB APOE ε3	**3.352 [0.034–47.453][a,b]**	**0.285 [0.001–7.846][a]**	**0.811 [0.007–14.81][a]**	**6 [0–6][a]**	**2 [0–5][a]**	**2 [0–6][a]**	**1 [0–3][a]**	14/70 (20%)
PDD/DLB APOE ε4	**5.864 [1.046–34.998][a,b,c]**	**2.397 [0.02–10.623][a,b,c]**	**0.761 [0.039–18.803][a]**	**6 [5–6][a,b,c]**	**3 [0–5] [a,b,c]**	**2 [1–6] [a,b]**	**1 [0–3] [a,b,c]**	5/28 (18%)

Values are mean ± SD for age at onset, disease duration and age at death. Age at onset was calculated from reported symptom onset. Disease duration is symptom onset to death. Lewy body density, % area of Aβ (%Aβ), and % area of pTau (%pTau) are quantitative averages across defined cortical and medial temporal regions. Values for Lewy body density, %Aβ, and %pTau, α-synuclein Braak stage, Aβ Thal phase, pTau Braak & Braak stage, and Alzheimer's disease neuropathological change (ADNC) National Institute on Aging-Alzheimer's Association (NIA-AA) levels are median with [range]. ADNC: 0-3; Braak stage: 0-6; Aβ Thal: 0-5; pTau Braak & Braak stage: 0-VI (numbered 0-6). TDP-43 LATE staging represented as % cases, since all positive cases correspond to stage 2. *P*-values Benjamini–Hochberg adjusted using Fisher's exact tests, χ^2 proportion tests, or pairwise Mann–Whitney tests as appropriate. Full statistics are provided in Supplementary Statistics Tables.

Bold text and superscripts indicate significant pairwise differences and correspond to the following contrasts:
[a] $P \leq 0.05$ vs PD APOE ε3,
[b] $P \leq 0.05$ vs PD APOE ε4,
[c] $P \leq 0.05$ vs PDD/DLB APOE ε3.

Supplementary Table 5. Clinical and demographic characteristics of Lewy body disease cohorts stratified by TDP-43 LATE stage.

Cohort	n	Male, n (%)	Age at onset, y	Disease duration, y	Age at death, y	APOE ε4 carriers, n (%)	Dementia, n (%)	Orthostatic hypotension, n (%)
TDP-43 LATE stage 0	232	134/232 (58%)	59.9 ± 11.5	17.0 ± 9.2	78.4 ± 8.0	41/176 (23%)	100/232 (43%)	84/121 (69%)
TDP-43 LATE stage 2	34	21/34 (62%)	62.4 ± 12.6	18.1 ± 9.7	**81.7 ± 7.9***	7/30 (23%)	21/34 (62%)	**7/20 (35%)***

Cohort	Lewy body density (LB/mm^2)	%Aβ	%pTau	α-synuclein Braak stage[18]	Aβ Thal phase[19]	pTau Braak & Braak stage[1]	ADNC[4, 5]	Ischaemic pathology, n (%)
TDP-43 LATE stage 0	1.476 [0.002–34.998]	0.28 [0.001–11.205]	0.791 [0.005–18.803]	6 [0–6]	2 [0–5]	2 [0–6]	1 [0–3]	0/232 (0%)
TDP-43 LATE stage 2	3.83 [0.003–47.453]	0.892 [0.001–10.623]	**2.569 [0.011–19.313]***	6 [0–6]	3 [0–5]	**2 [0–6]***	1 [0–3]	34/34 (100%)

Values are mean ± SD for age at onset, disease duration and age at death. Age at onset was calculated from reported symptom onset. Disease duration is symptom onset to death. Lewy body density, % area of Aβ (%Aβ), and % area of pTau (%pTau) are quantitative averages across defined cortical and medial temporal regions. Values for Lewy body density, %Aβ, and %pTau, α-synuclein Braak stage, Aβ Thal phase, pTau Braak & Braak stage, and Alzheimer's disease neuropathological change (ADNC) National Institute on Aging-Alzheimer's Association (NIA-AA) levels are median with [range]. ADNC: 0-3; Braak stage: 0-6; Aβ Thal: 0-5; pTau Braak & Braak stage: 0-VI (numbered 0-6). TDP-43 LATE staging represented as % cases, since all positive cases correspond to stage 2. *P*-values are Benjamini–Hochberg corrected values based on Fisher's exact tests, χ^2 proportion tests, or pairwise Mann–Whitney tests, as appropriate. Full statistics are provided in Supplementary Statistics Tables.
Bold text and superscripts indicate significant pairwise differences and correspond to the following significant contrast:
* $P<0.05$ for TDP-43 LATE stage 0 vs TDP-43 LATE stage 2

Supplementary Table 6. Measurements collected for each pseudo-cell in Lewy body classifier.

Feature	Pseudo-cell compartment	Measurement
Intensity	Nucleus (original detection object), cytoplasm (2 μm background around detection object), cell (nucleus + cytoplasm)	Mean, median, min, max, and standard deviation for red, green, blue, deconvoluted haematoxylin, deconvoluted DAB, and deconvoluted residual channels.
Shape/morphology	Nucleus (original detection object)	Area, length, circularity, solidity, max diameter, min diameter.
Haralick[20]	Nucleus (original detection object)	Angular second moment (F0), contrast (F1), correlation (F2), sum of squares (F3), inverse difference moment (F4), sum average (F5), sum variance (F6), sum entropy (F7), entropy (F8), difference variance (F9), difference entropy (F10), information measure of correlation 1 (F11), information measure of correlation 2 (F12).

References

1. Braak H, Braak E. Neuropathological stageing of Alzheimer-related changes. Acta neuropathologica 1991;82:239-259.
2. Thal DR, Rüb U, Orantes M, Braak H. Phases of A beta-deposition in the human brain and its relevance for the development of AD. Neurology 2002;58:1791-1800.
3. Braak H, Del Tredici K, Rüb U, de Vos RA, Jansen Steur EN, Braak E. Staging of brain pathology related to sporadic Parkinson's disease. Neurobiology of aging 2003;24:197-211.
4. Hyman BT, Phelps CH, Beach TG, et al. National Institute on Aging-Alzheimer's Association guidelines for the neuropathologic assessment of Alzheimer's disease. Alzheimer's & dementia : the journal of the Alzheimer's Association 2012;8:1-13.
5. Montine TJ, Phelps CH, Beach TG, et al. National Institute on Aging-Alzheimer's Association guidelines for the neuropathologic assessment of Alzheimer's disease: a practical approach. Acta neuropathologica 2012;123:1-11.
6. Nelson PT, Dickson DW, Trojanowski JQ, et al. Limbic-predominant age-related TDP-43 encephalopathy (LATE): consensus working group report. Brain : a journal of neurology 2019;142:1503-1527.
7. McKeith IG, Boeve BF, Dickson DW, et al. Diagnosis and management of dementia with Lewy bodies: Fourth consensus report of the DLB Consortium. Neurology 2017;89:88-100.
8. Bankhead P, Loughrey MB, Fernández JA, et al. QuPath: Open source software for digital pathology image analysis. Scientific reports 2017;7:16878.
9. Schmidt U, Weigert M, Broaddus C, Myers G. Cell Detection with Star-Convex Polygons. In: Frangi AF, Schnabel JA, Davatzikos C, Alberola-López C, Fichtinger G, ed. Medical Image Computing and Computer Assisted Intervention – MICCAI 2018; 2018 2018//; Cham: Springer International Publishing: 265-273.
10. Zack GW, Rogers WE, Latt SA. Automatic measurement of sister chromatid exchange frequency. The journal of histochemistry and cytochemistry : official journal of the Histochemistry Society 1977;25:741-753.
11. Otsu N. A Threshold Selection Method from Gray-Level Histograms. IEEE Transactions on Systems, Man, and Cybernetics 1979;9:62-66.
12. R: A Language and Environment for Statistical Computing [computer program]. Vienna, Austria: R Foundation for Statistical Computing, 2024.
13. Robin X, Turck N, Hainard A, et al. pROC: an open-source package for R and S+ to analyze and compare ROC curves. BMC Bioinformatics 2011;12:77.
14. Youden WJ. Index for rating diagnostic tests. Cancer 1950;3:32-35.
15. Young AL, Marinescu RV, Oxtoby NP, et al. Uncovering the heterogeneity and temporal complexity of neurodegenerative diseases with Subtype and Stage Inference. Nature communications 2018;9:4273.
16. Vogel JW, Young AL, Oxtoby NP, et al. Four distinct trajectories of tau deposition identified in Alzheimer's disease. Nature medicine 2021;27:871-881.
17. Benjamini Y, Hochberg Y. Controlling the False Discovery Rate: A Practical and Powerful Approach to Multiple Testing. Journal of the Royal Statistical Society: Series B (Methodological) 2018;57:289-300.
18. Braak H, Del Tredici K, Rub U, de Vos RA, Jansen Steur EN, Braak E. Staging of brain pathology related to sporadic Parkinson's disease. Neurobiol Aging 2003;24:197-211.
19. Thal DR, Rub U, Orantes M, Braak H. Phases of A beta-deposition in the human brain and its relevance for the development of AD. Neurology 2002;58:1791-1800.

20. Haralick RM. Statistical and structural approaches to texture. Proceedings of the IEEE 1979;67:786-804.
